# Semi-dwarfism and lodging tolerance in tef (*Eragrostis tef*) is linked to a mutation in the *α-Tubulin 1* gene

**DOI:** 10.1093/jxb/eru452

**Published:** 2014-11-15

**Authors:** Moritz Jöst, Korinna Esfeld, Agata Burian, Gina Cannarozzi, Solomon Chanyalew, Cris Kuhlemeier, Kebebew Assefa, Zerihun Tadele

**Affiliations:** ^1^Institute of Plant Sciences, University of Bern, Altenbergrain 21, CH-3013 Bern, Switzerland; ^2^Ethiopian Institute of Agricultural Research, Debre Zeit Agricultural Research Center, PO Box 32, Debre Zeit, Ethiopia

**Keywords:** *Ergarostis tef*, *kegne*, lodging, microtubules, semi-dwarf, tef, *α-Tubulin*.

## Abstract

Genetic improvement of native crops is a new and promising strategy to combat hunger in the developing world. Tef is the major staple food crop for approximately 50 million people in Ethiopia. As an indigenous cereal, it is well adapted to diverse climatic and soil conditions; however, its productivity is extremely low mainly due to susceptibility to lodging. Tef has a tall and weak stem, liable to lodge (or fall over), which is aggravated by wind, rain, or application of nitrogen fertilizer. To circumvent this problem, the first semi-dwarf lodging-tolerant tef line, called *kegne*, was developed from an ethyl methanesulphonate (EMS)-mutagenized population. The response of *kegne* to microtubule-depolymerizing and -stabilizing drugs, as well as subsequent gene sequencing and segregation analysis, suggests that a defect in the *α-Tubulin* gene is functionally and genetically tightly linked to the *kegne* phenotype. In diploid species such as rice, homozygous mutations in *α-Tubulin* genes result in extreme dwarfism and weak stems. In the allotetraploid tef, only one homeologue is mutated, and the presence of the second intact *α-Tubulin* gene copy confers the agriculturally beneficial semi-dwarf and lodging-tolerant phenotype. Introgression of *kegne* into locally adapted and popular tef cultivars in Ethiopia will increase the lodging tolerance in the tef germplasm and, as a result, will improve the productivity of this valuable crop.

## Highlight

The semi-dwarf and lodging-tolerant *kegne* mutant linked to defects in microtubule orientation has the potential to enhance the productivity of an African orphan crop tef (*Eragrostis tef*).

## Introduction

Tef [*Eragrostis tef* (Zucc.) Trotter] belongs to the family of Poaceae (subfamily Chloridoideae) and is a C_4_, self-pollinated, and allotetraploid plant species. As the most important cereal crop in Ethiopia, tef is cultivated annually on 2.8 million ha of land, ~29% of the area allocated to cereals (CSA, 2011). Tef is a resilient crop that performs better than other cereals under local conditions including drought, waterlogging, and poor soil. Since it produces a reasonable yield when grown in areas that experience moisture scarcity, it is considered as a low risk crop (Ketema, 1997). Tef is nutritious due to its high protein and mineral content (Bultosa *et al.*, 2002; Abebe *et al.*, 2007), and the absence of gluten (Spaenij-Dekking *et al.*, 2005) makes it an alternative food for people suffering from coeliac disease.

Despite its versatility in adapting to adverse environmental conditions and being the staple food for ~50 million people in the Horn of Africa, seed yield of tef is low. The national average yield is ~1.3 t ha^–1^, in contrast to 3.0 t ha^–1^ for rice (CSA, 2011). Provided with optimal fertilizer, bird protection, and a mesh to prevent lodging, a yield of 3.4–4.6 t ha^–1^ could be achieved (Teklu and Tefera, 2005). Nevertheless, such agricultural inputs are expensive and time-consuming, and therefore not desirable for agricultural practice. A major cause of low productivity of tef is lodging, the permanent displacement of the stem from the upright position. Tef has a tall and slender stem which is susceptible to lodging caused by wind and rain. In addition, when fertilizer is applied to increase yield, stems of tef grow taller and become even more susceptible to lodging, resulting in significantly reduced quantity and quality of grain and straw. Moreover, lodging makes harvesting by hand difficult and mechanical harvesting nearly impossible. The average yield reduction due to lodging is estimated at 17% (Ketema, 1993).

Major yield improvements in rice and wheat were achieved in the 1960s through intensive breeding, known as the ‘Green Revolution’. One important trait of these improved varieties was their semi-dwarf phenotype, which resulted in increased standing ability and resource reallocation into grain rather than shoot biomass (Evenson and Gollin, 2003). Plant-specific hormones such as gibberellic acid (GA), brassinosteroid (BR), and auxin, as well as their signalling pathways are known to regulate plant height (Wang and Li, 2008). Likewise, microtubules have also been proven to control this trait (Sunohara *et al.*, 2009).

Mutations in GA biosynthesis genes lead to lower internal concentrations of GA, resulting in dwarf phenotypes in both monocots and dicots. The altered plants are often impaired in internode elongation, having dark green leaves and shorter hypocotyls, but these phenotypes can be rescued by application of exogenous GA (Itoh *et al.*, 2004; Rieu *et al.*, 2008). Mutants with defects in GA signalling have a similar phenotype but, in contrast, are insensitive to GA treatments (Gale *et al.*, 1975; Dill *et al.*, 2001; Ueguchi-Tanaka *et al.*, 2005). In both cases, the reduced height and short hypocotyls are probably due to a reduction in cell elongation (Cowling and Harberd, 1999).

Brassinosteroids are also important regulators of plant development and cell elongation, and can promote plant growth (Fujioka and Yokota, 2003; Tanaka *et al.*, 2003; Bajguz and Asami, 2004, 2005). Many mutants deficient in BR biosynthesis or in BR signalling have been identified in both dicots and monocots. The majority of these mutants have a short stature, short roots, and erect and dark green leaves. One important character common in these mutants is the de-etiolated growth in the dark due to defects in cell elongation. BR-deficient mutants can be rescued by exogenous application of brassinolide (BL), a bioactive BR, whereas BR signalling mutants are insensitive to exogenously applied BL (Szekeres *et al.*, 1996; Noguchi *et al.*, 1999; Choe *et al.*, 2002; Chono *et al.*, 2003; Hong *et al.*, 2003; Sakamoto *et al.*, 2006; Jager *et al.*, 2007).

A third hormone known to regulate plant height is auxin (Taiz and Zeiger, 2006). Mutations leading to alterations in auxin transport and subsequently dwarfism were reported in maize [*brachytic2* (*br2*)], sorghum [*dwarf3* (*dw3*)], and *Arabidopsis* [*twisted dwarf 1* (*twd1*)] (Multani *et al.*, 2003; Bouchard *et al.*, 2006). Other auxin-related mutants are insensitive to exogenously applied auxin and experience an altered gravitropic response of the roots (Hobbie and Estelle, 1995; Lewis *et al.*, 2007).

Recently, microtubules were also proved to regulate plant height, since *Arabidopsis* mutants affected in microtubule organization were dwarf in stature (Wang and Li, 2008). Together with actin filaments, microtubules are key components for many processes in plant growth and development, including cell division, cell differentiation, cell to cell communication, and cell expansion. In plants, cellulose microfibrils, which are important constituents of the extracellular matrix, orient along microtubules. Generally, during anisotropic cell expansion, cellulose microfibrils are arranged parallel to each other and oriented transversely to the direction of growth. This leads to increased radial resistance to the turgor pressure and cell expansion perpendicular to microtubule orientation. In addition, many microtubule-associated proteins (MAPs) are particularly important for cross-linking, orientation, and organization of microtubules (Kost *et al.*, 1999; Lloyd and Chan, 2004). Microtubules are made up of α-/β-tubulin heterodimers which form protofilaments through longitudinal arrangements (Nogales *et al.*, 1999; Lowe *et al.*, 2001). Microtubules are very dynamic elements that are characterized by a continuous turnover of subunits undergoing polymerization at the plus end and depolymerization at the minus end (Shaw *et al.*, 2003). Mutations related to microtubules affect the architecture of plants. For instance, a non-synonymous amino acid substitution in an *α-Tubulin gene* created the *twisted dwarf 1* (*tid1*) mutant of rice, which also showed helical growth of leaves (Sunohara *et al.*, 2009). In *Arabidopsis*, mutations in the homologous gene led to two *lefty* mutants, which were mainly characterized by the helical growth of aerial organs and their short skewing roots. However, an effect of the *lefty* mutations on plant height was not reported (Thitamadee *et al.*, 2002; Ishida *et al.*, 2007*a*). Similar phenotypes have been observed in further *Arabidopsis* mutants such as *spiral 1* (*spr1*) and *tortifolia 1* (*tor1*) that carry mutations in MAPs (Furutani *et al.*, 2000; Buschmann *et al.*, 2004). All *Arabidopsis* mutants indicated above experienced increased sensitivity to the microtubule-depolymerizing drug propyzamide and the microtubule-stabilizing drug taxol (Furutani *et al.*, 2000; Thitamadee *et al.*, 2002; Buschmann *et al.*, 2004; Shoji *et al.*, 2004). Apart from showing a helical phenotype, amino acid alterations in α-tubulin proteins of some Poaceae species (e.g. *Setaria viridis* and *Eleusine indica*) confer a resistance to dintroaniline (commonly known as oryzalin), the active compounds of several non-selective herbicides (Yamamoto *et al.*, 1998; Yamamoto and Baird, 1999; Delye *et al.*, 2004).

In this study, a novel tef mutant named *kegne*, which has a semi-dwarf phenotype, resulting in increased lodging tolerance, is characterized. Besides their short stature, *kegne* plants show right-handed twisting in young leaves and dark-grown coleoptiles as well as reduced cell sizes. Microtubule drug assays and microtubule labelling with anti-tubulin antibody indicated that the mutant phenotype is likely to be caused by a mutation in an *α-Tubulin* gene and prevailing left-handed microtubule arrays in epidermal cells. A point mutation was identified in the *α-Tubulin 1* gene which co-segregates with the *kegne* phenotype and serves as a marker for marker-assisted breeding.

## Materials and methods

### Chemicals

All chemicals were purchased from Sigma-Aldrich, Switzerland except brassinozole220 (Brz220), an inhibitor of BR biosynthesis (Asami *et al.*, 2000; Sekimata *et al.*, 2008), which was a kind gift of Dr Tadao Asami (University of Tokyo). BL, GA, and Brz220 were dissolved in 96% ethanol. *N*-1-naphthylphthalamic acid (NPA), indole-3-acetic acid (IAA; auxin), paclobutrazol, propyzamide, taxol (paclitaxel), and oryzalin were dissolved in dimethylsulphoxide (DMSO).

### Plant material and growth conditions

The *kegne* mutant was derived from an EMS (ethyl methanesulphonate)-mutagenized population of the tef cultivar DZ-Cr-37 [*Tsedey*, hereafter called the wild type (WT)], generated for the ‘Tef Improvement Project’ at the University of Bern. The EMS concentration used for the mutagenesis of the tef seeds was 0.2% (v/v).

The F_2_ progeny of crosses between *kegne* and three improved varieties, namely *Magna* (cross name, MK), *Quncho* (QK), and *Tsedey* (TK), as well as the ecotype *Kay muri* (KK) were investigated.

For plants grown on soil, a mix of 50% topsoil, 40% peat, and 10% sand was used. The plants were fertilized with compound fertilizer (N:P:K, 2.7:1:4.3 and trace elements). Growth conditions were either short day (8h light at 22 °C and 16h dark at 20 °C), equal day (12h light at 24 °C and 12h dark at 18 °C), or long day conditions (16h light at 22 °C and 8h dark at 20 °C) with 65–75% relative humidity at all light regimes.

For experiments under *in vitro* conditions, seeds were surface sterilized for 5min using 1% (v/v) sodium hypochlorite solution plus a drop of Tween-20 as wetting agent. Following sterilization, seeds were rinsed 3–4 times with distilled water and sown on square plates with half-strength Murashige and Skoog (1/2 MS) salts, 1% (w/v) sucrose, and 1% agar. The indicated amounts of hormones were added to the autoclaved and cooled media (50–60 °C). Control plates received DMSO or ethanol in the same amounts as those with treatments. Plants were grown vertically for the indicated number of days in dark or light, in a growth chamber with 14h of light at 25 °C and 10h of dark at 21 °C. For height- or length-related measurements, plates were scanned and the pictures were analysed using the software ImageJ (NIH, USA). For the oryzalin experiments, plants were transferred after 10 d from oryzalin-containing media to 1/2 MS media, containing no oryzalin, and grown for a further 6 d. Plant height was measured after 10 d and 16 d.

### Cell size and twisting measurements

For cell length measurements, coleoptiles of 16 plants per tef line, grown for 4 d in the light and 3 d in the dark under *in vitro* conditions, were imaged with a scanning electron microscope (SEM). The length of 15 individual cells per plant was measured. Under dark conditions, the length of six coleoptile cells of two individual plants was measured. In order to measure the twisting angle, 3-day-old, dark-grown coleoptiles were imaged with the SEM. The angle of twisting relative to both edges of the coleoptile was measured using the angle tool in ImageJ. For each line, 12 coleoptiles were imaged and the twisting angle of eight cells for every coleoptile was measured.

For F_2_ plants, seeds were grown under *in vitro* conditions in the dark for 3 d. Pictures of dark-grown coleoptiles were taken with a digital microscope (VHX-600, Keyence, Germany) and analysed using ImageJ. Plants were grouped into *kegne*, WT, and heterozygous based on the helical growth of the coleoptiles. After taking pictures, plants were collected in liquid nitrogen and DNA was extracted for each plant. PCR amplification and restriction digestion of the *Eragrostis tef α-Tubulin 1* (*EtTUA1*) gene was performed as described below.

### Plant height and yield components

For weekly plant height measurements, 10 plants each from the WT and *kegne* were grown on soil under equal day conditions. Physiologically mature plants were harvested 105 d and 127 d after sowing for the WT and *kegne* mutant, respectively. Plants were dried at 40 °C for 10 d before biomass and seed yield were measured. The harvest index was calculated as the ratio of seed yield to the above-ground biomass. For the yield experiment, plants of the WT and *kegne* mutant were grown under equal day conditions in a tray (45.5×28.2×6.0cm) filled with soil. Seeds were sown in lines 2cm apart. After germination, plants were thinned out to exactly 312 plants per tray, with ~2cm space between each individual plant. Sixteen weeks after sowing, all plants were harvested and dried at 40 °C for 10 d before biomass and seeds were weighed. The harvest index was calculated as mentioned above. F_2_ plants were grown on individual pots for the first 3–4 weeks under long day conditions followed by short day conditions until the panicles fully emerged.

### RNA and DNA extraction

RNA extraction was done using the Trizol method (Invitrogen). M-MLV transcriptase (Promega) was used for cDNA synthesis. Genomic DNA extraction was done either with the NucleoSpin 96 Plant extraction kit (Machery-Nagel, Germany) following the protocol of the supplier or by grinding the leaf material with a mill (MM 300 Retsch, Germany) before adding 200 μl of extraction buffer containing 200mM TRIS-HCl (pH 7.5), 250mM NaCl, 25mM EDTA (pH 8.0), and 0.5% SDS. Afterwards, a chloroform/isopropanol purification was performed before the extracted DNA was treated with 3 μl of a 10mg ml^–1^ RNase A solution.

### Amplification and sequencing of *α-Tubulin* genes

cDNA was used for amplification of the orthologous *α-Tubulin* genes from tef. Primers were designed using sequence information of the *α-Tubulin* genes of *E. indica* (the respective accession numbers for *TUA1*, *TUA2*, and *TUA3* are AF008120, AF008121, and AF008122). For each of the three genes, two sets of primers were designed; one pair at the 5’ (S1) and 3’ end (AS1) and the other set in the central region of the gene (S2 and AS2). In addition, a pair of primers was designed to amplify specifically copy A of the genomic *EtTUA1* gene. The sequences of the primers are indicated below: Tua1S1, ACCATGAGGGAGTGCATCTCGAT; Tua1AS1, ACCCAGTAGCAAAGCGGGGAATT; Tua1S2, ATTG AGCGCCCAACCTACACCAA; Tua1AS2, AGCATGAAGTGGA TCCTCGGGTA; Tua1S copy A, CTGAGCCAAGTTCGTTGATT GCTT; Tua2S, TGGCTCTGGACTTGGTTCACTAC; Tua2AS1, TACTCTGCCAGCCATGAACAGGT; Tua2S2, TGCTGCAGGC CTTTTCAGTTCTC; Tua2AS2, CAGAGTGCTGTTCATGGTAG GCT; Tua3S1, TTCAACACCTTCTTCAGCGAGAC; Tua3S2, CTTGAACAGGCTGATCTCACAGA; and Tua3AS1, ACAATC ATGACCAACAGGGGGCT.

For sequencing, the amplified PCR products were ligated with the pGEM^®^-T easy vector system (Promega) with procedures performed as outlined in the Quick protocol (www.promega.com) and DH5α as competent cells were transformed. Sequencing of the inserts was done from purified plasmids. The respective amplicons were sequenced and used to develop gene copy-specific primers used on genomic DNA.

Genomic DNA was used as template in a PCR to amplify the *TUA1* gene for CAPS (cleaved amplified polymorphic sequence) marker assays. In order to score F_2_ plants as homozygous wild type, heterozygous, or homozygous *kegne*, the PCR-amplified products for the copy-specific *EtTUA1* gene were digested using the restriction enzyme *Cla*I (New England Biolabs). PCR was performed in a 10 μl volume using 1× buffer, 0.2mM dNTPs, 0.325 μM of each forward (Tua1S copy A) and reverse (Tua1AS1) primers, and 0.1U of GoTaq polymerase (Promega) using 33 cycles of 95 °C for 50 s, 59 °C for 50 s, and 72 °C for 2min. The amplified PCR products were evaluated on agarose gels after incubating with 2.5–5U of *Cla*I.

F_3_ progeny of crosses between *kegne* and three improved tef cultivars were investigated to confirm the co-segregation between *kegne*-related phenotypes and the *EtTUA1* marker.

### Microtubule immunolabelling

Five-day-old seedlings grown in the dark on 1/2 MS plates were fixed in 1.5% formaldehyde and 0.5% glutaraldehyde in microtubule-stabilizing buffer (MTSB; 50mM PIPES, 2mM EGTA, 2mM MgSO_4_, pH 7.2) with 0.05% Triton X-100 (v/v) and 5% DMSO for 1h, and rinsed three times for 10min in MTSB. Fixed seedlings were incised (usually epidermis and 2–3 layers underneath) with razor blades to enable diffusion of antibodies into cells. Samples were then incubated with primary antibody [monoclonal anti-α-tubulin, Sigma-Aldrich, diluted 1:100 in 1% bovine serum albumin (BSA) in phosphate-buffered saline (PBS)] for 2–3h in 37°C, rinsed twice for 10min in PBS, and incubated with secondary antibody [anti-mouse IgG–fluorescein isothiocyanate (FITC), Sigma-Aldrich, diluted 1:100 in 1% BSA in PBS] for 2–3h in 37 °C. After rinsing twice for 10min in PBS, the whole seedling was mounted in 1:1 PBS–glycerol with 1% *p*-phenylenediamine (Sigma-Aldrich). Images of cortical microtubules under the outer epidermal cell wall of a coleoptile were collected using a Zeiss (Axioskop2) fluorescent microscope with ×100 oil immersion objective.

Six and five seedlings were analysed for the WT and *kegne* mutant, respectively. In a cell, the orientation of cortical microtubules was measured using ImageJ software with a plug-in to quantify microtubule orientation (Boudaoud *et al.*, 2014). Microtubule orientation was defined with regard to the long cell axis. Transverse orientation refers to microtubules arranged between 80 ° and 100 ° to the cell axis. While microtubules oriented at an angle <80 ° are left-handed, those at an angle >80 ° are right-handed. The mean microtubule orientation and standard deviation (SD) were computed using circular statistics (Zar, 1999). Microtubule measurements were taken from a cell wall section of a length at least that of the cell width (one patch per cell). Angular distributions were compared using the non-parametric Kolmogorov–Smirnov test (K–S test) at the 0.05 level using the Origin software (OriginLab Corporation, USA).

## Results

### Phenotype of *kegne* plants

**Fig. 1.**
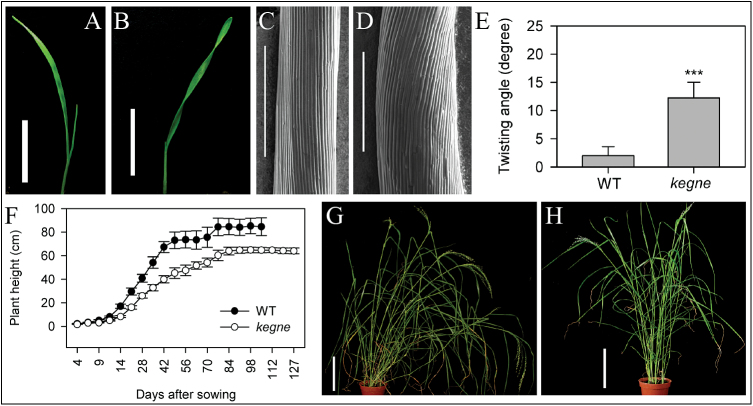
*Kegne* plants twist in the right-hand direction, and are semi-dwarf and lodging tolerant. Nine-day-old shoots of (A) WT and (B) *kegne*, and 3-day-old coleoptiles of (C) WT and (D) *kegne* in a growth room. (E) The angle of tilting was significantly higher for *kegne* compared with the WT (*P*=1.83 E-76 after ANOVA, *n*=96, error bars indicate 1 SD). (F) The height of both genotypes from germination to harvest maturity; error bars indicate 1 SD (*n*=10). Two-month-old (G) WT and (H) *kegne* plants in the greenhouse. Scale bars in A and B=1cm, in C and D=500 μM, in G and H=10cm.

A tef mutant with a helical phenotype was identified from an EMS-mutagenized M_2_ population of the improved tef cultivar DZ-Cr-37 (also known as *Tsedey*; here referred to as the WT). The mutant was named *kegne*, meaning ‘right hand’ in the Ethiopian Amharic language, for the unique turning of its first leaves towards the right-hand direction (Fig. 1A, B). The helical phenotype of *kegne* was also pronounced in dark-grown coleoptiles (Fig. 1C, D), with cells tilting off the vertical axis on average by ~12 ° compared with only 2 ° in the WT (Fig. 1E). Strikingly, the cells of *kegne* coleoptiles twisted in all cases in the right-hand (or clock-wise) direction, whereas those in the WT tilted off the central axis in either orientation.

*Kegne* plants are also semi-dwarf in height. They were shorter than the WT throughout the growing period (Fig. 1F). At physiological maturity, *kegne* plants were ~25% shorter than the WT. However, *kegne* reached harvest maturity ~10 d later than the WT. The helical growth of *kegne* leaves became less pronounced at later developmental stages, although the erect phenotype of the plant was still evident (Fig. 1G, H).

**Fig. 2.**
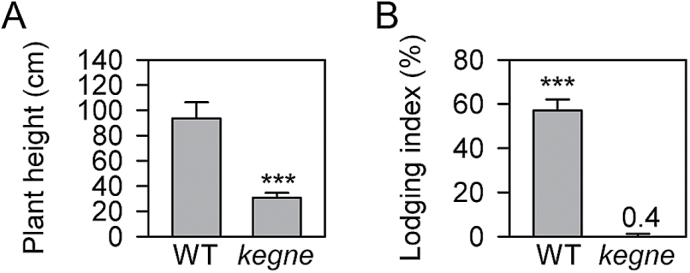
Plants grown in the field in Ethiopia. (A) Plant height and (B) lodging index of the WT compared with the *kegne* mutant at maturity (*n*=30, height *P*=1.70E-33, lodging *P*=2.43E-54 after ANOVA, error bars indicate 1 SD).

Field tests in Ethiopia indicated that the *kegne* mutant attained only one-third of the height of the WT (Fig. 2A) and showed no tendency to lodge, unlike the original line that had an average of 60% lodging (Fig. 2B).

Similar to the WT, coleoptiles of *kegne* plants showed de-etiolated growth when grown in the dark, but attained only half the size of the WT (Supplementary Fig. S1A available at *JXB* online). Cell length measurements showed that both light- and dark-grown coleoptiles of *kegne* were significantly shorter than those of the WT (Supplementary Fig. S1B).

### Hormone assays

Since phytohormones such as GA, BR, and auxin are known to regulate cell elongation and plant height, the responses of *kegne* to these hormones and their respective inhibitors were investigated. WT and *kegne* plants were grown in either the light or the dark for 3 d on 1/2 MS medium containing different concentrations of GA. The unique phenotype of *kegne* plants in dark and light conditions was not rescued to the WT level by GA application (Supplementary Fig. S2 at *JXB* online), suggesting that *kegne* is not impaired in the biosynthesis of GA. When grown for 29 d on GA-containing medium, a significant increase in the length of both shoots and leaf sheaths was observed for both genotypes (data not shown). Although the same height as the WT was not achieved, *kegne* plants were responding to a certain degree to GA application, indicating that the mutation in *kegne* is not due to GA insensitivity.

The effects of different levels of paclobutrazol, a GA biosynthesis inhibitor, were also investigated on *in vitro* grown plants. WT shoots under both dark and light and roots under light conditions were significantly inhibited by increasing levels of paclobutrazol. In contrast, *kegne* plants were unaffected by paclobutrazol application, except for the shoots grown in light (Supplementary Fig. S3 at *JXB* online).

Similar to GA, the application of different levels of BL did not rescue *kegne* plants to the WT level. On the other hand, high BL concentrations led to a significant decrease in plant height and root length for both genotypes grown in the light and dark, respectively. This indicates that *kegne* is probably not impaired in the biosynthesis of BR but is responsive to BL application (Supplementary Fig. S4 at *JXB* online).

Nevertheless, BR might induce the etiolated growth of tef in the dark, as evidenced by the application of Brz220, an inhibitor of BR biosynthesis. Both the WT and *kegne* plants showed de-etiolated growth when grown on high concentrations of Brz220 in the dark, although the WT was more sensitive to the treatments (Supplementary Fig. S5 at *JXB* online).

In addition, auxin and NPA, a polar auxin transport inhibitor, did not significantly affect the growth of roots and shoots in both genotypes (data not shown). Since defects in auxin signalling normally result in an altered gravitropic response of the roots, a root-bending assay was performed, although no obvious alterations were detected between the two genotypes (data not shown).

In general, none of the hormones and inhibitors substantially altered the response of *kegne* plants, indicating that the mutant is not impaired in either the biosynthesis or signalling of these three phytohormones.

### Tubulin drug assays

Mutations in microtubule-associated genes in rice and *Arabidopsis* lead to dwarfism and helical shoot growth (Ishida *et al.*, 2007*a*; Sunohara *et al.*, 2009). To investigate whether the *kegne* phenotype is due to a defect associated with microtubules, the responses to taxol, a microtubule-stabilizing drug, and to propyzamide, a microtubule-depolymerizing drug, were tested. Both the WT and *kegne* plants were affected in a similar way by taxol (data not shown). However, the two genotypes differed in their response to propyzamide (Supplementary Fig. S6 at *JXB* online). In contrast to the WT, *kegne* shoots grown in the dark were not reduced in length at 1 μM propyzamide. Only at higher concentrations were both genotypes affected. The roots of *kegne* were insensitive to increasing levels of propyzamide in both light regimes, except for the highest concentration tested.

**Fig. 3.**
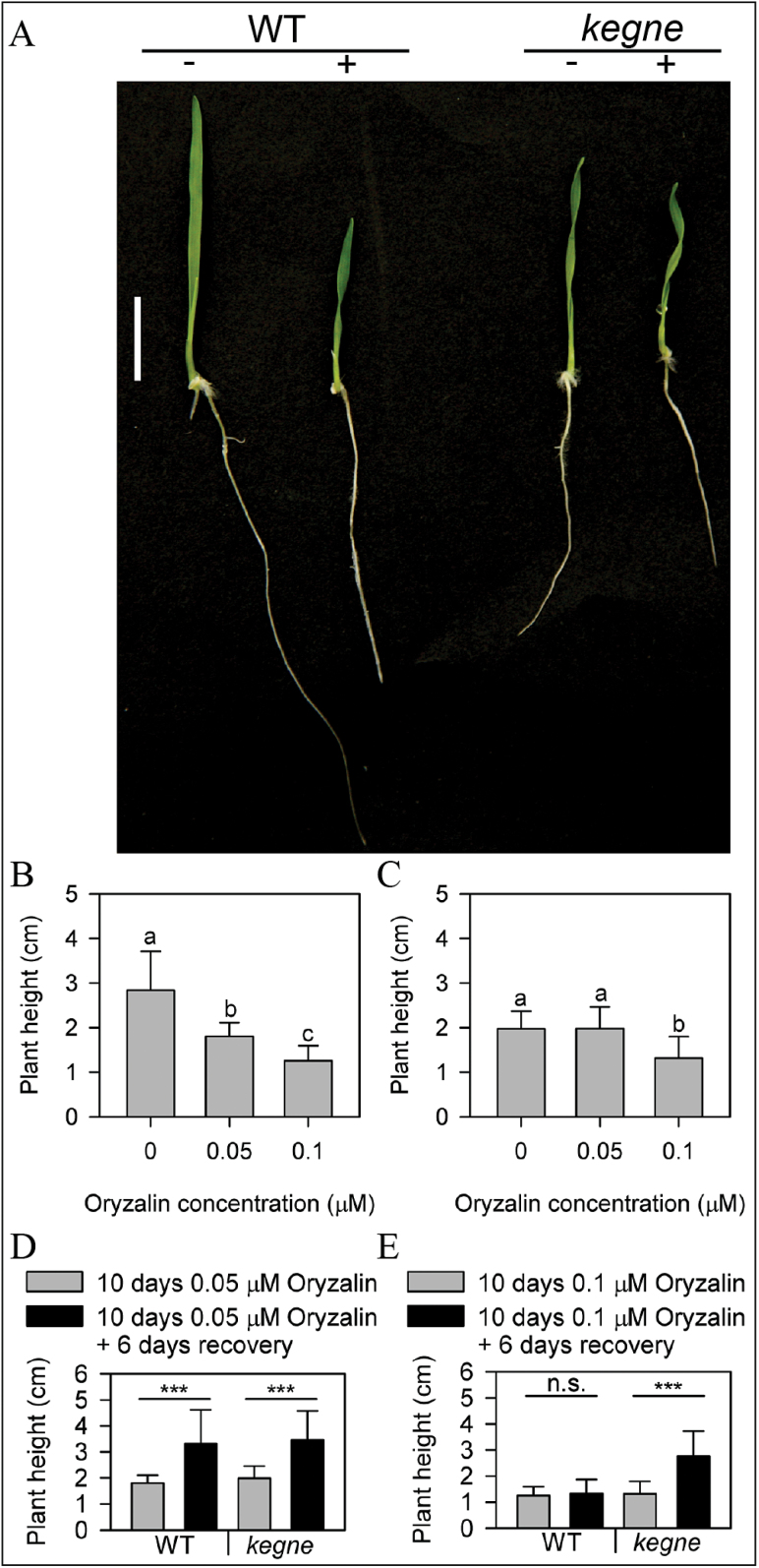
The response of *kegne* to propyzamide and oryzalin. (A) The twisting phenotype of *kegne* was induced in 3-day-old WT plants treated with 2 μM propyzamide. Scale bar=1cm. Effect of different concentrations of oryzalin on the height of (B) WT and (C) *kegne* plants grown for 10 d in the light. Values with the same letters are not significantly different at the *P*<0.05 level after Tukey test (*n*=15). Error bars indicate 1 SD. The height of seedlings transferred to the medium without oryzalin and grown for an additional 6 d to recover from (D) 0.05 μM (*n*=15) and (E) 0.1 μM oryzalin (*n*=15).

Propyzamide also converted WT plants to the helical phenotype observed in the *kegne* mutant (Fig. 3A). The fact that twisting was induced in WT plants treated with a microtubule-destabilizing drug strongly suggests that this phenotype of *kegne* is caused by defects associated with microtubules.

Oryzalin is known to disrupt microtubule files by specifically binding to α-tubulin (Murthy *et al.*, 1994; Morrissette *et al.*, 2004). When grown for 10 d on an *in vitro* medium containing 0.05 μM oryzalin, WT plants became stunted (Fig. 3B). In contrast, *kegne* plants were affected only at 0.1 μM oryzalin (Fig. 3C). After transferring them to normal MS medium without the drug, WT plants resumed normal growth when treated with a maximum of 0.05 μM oryzalin while *kegne* plants recovered even from 0.1 μM oryzalin (Fig. 3D, E). The resistance of *kegne* to oryzalin suggests a mutation in an *α-Tubulin* gene.

### Microtubule immunolabelling

**Fig. 4.**
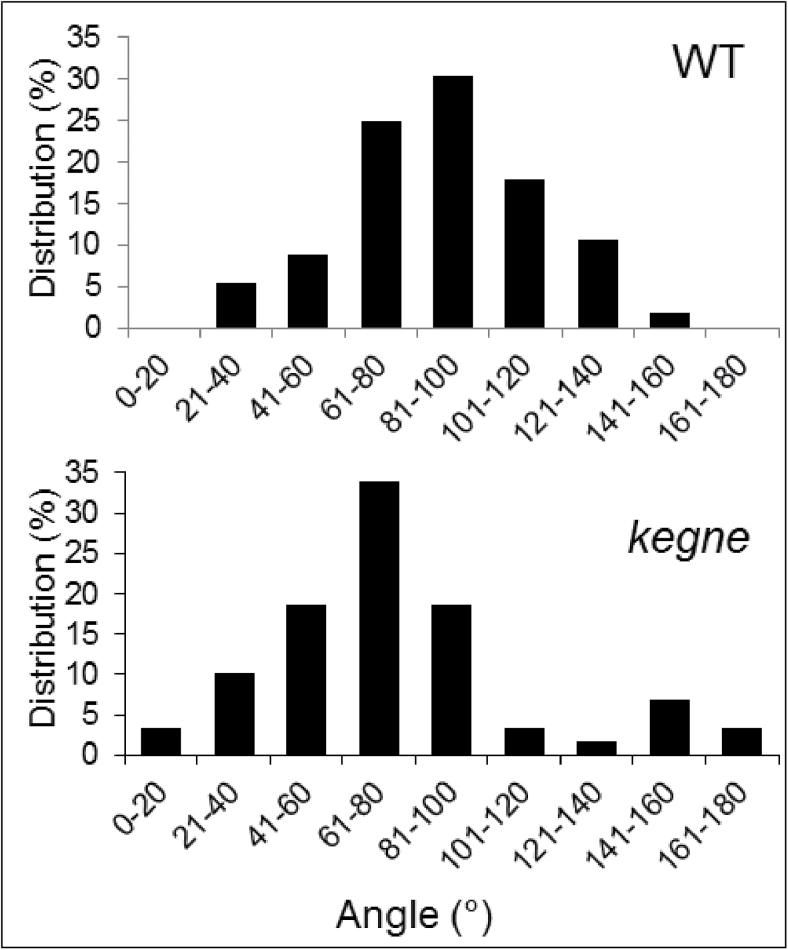
The orientation of cortical microtubules in coleoptiles of the WT and *kegne*. Distributions of microtubule orientations (in degrees) in epidermal cells (*n*=56 cells for the WT, and *n*=59 cells for *kegne*) of 5-day-old dark-grown seedlings.

Usually twisting mutants have microtubule arrays of a specific handedness; for example, in the mutants with right-handed twisting epidermis most microtubules form left-handed helices, and *vice versa* (Ishida *et al.*, 2007*b*). To check whether it is also the case in the right-handed twisting *kegne* mutant, the orientation of cortical microtubules under the outer epidermal cell wall of coleoptiles was measured in reference to the long cell axis. Microtubules were classified as transverse for orientation between 80 ° and 100 °, left-handed for those below 80 °, and right-handed for those above 100 °. In the WT, 30% of microtubules had transverse orientation (Fig. 4), although oblique orientations of either left- or right-handedness were also present. However, in the *kegne* mutant, the majority of microtubules were left-handed (Fig. 4). While the mean microtubule orientation was 88 ° for the WT (SD=0.96), it was 66 ° for the *kegne* mutant (SD=0.95). The statistical test confirmed that the angle distributions for the WT and *kegne* were significantly different (K–S test, *P*<0.05). This indicates that the microtubules in *kegne* were distorted compared with those in the WT.

### Mutation discovery through the candidate gene approach

**Fig. 5.**
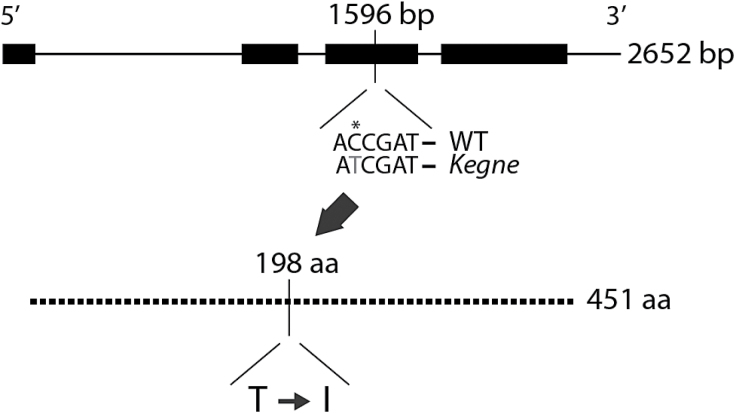
Overview of the structure of the *EtTUA1* gene and the position of the point mutation in a single genomic copy of the *kegne* mutant. Black boxes indicate the four exons while the lines show the three introns. The total size of the CDS of *EtTUA1* is 1353bp or 451 amino acids. The single mutation discovered in *kegne* was at 1596bp on the genomic sequence or 198 amino acids on the CDS. The C to T change in *kegne* introduced a unique site for the *Cla*I restriction enzyme which can be used as a CAPS marker and leads to an amino acid substitution from a threonine (T) in the WT to an isoleucine (I) in *kegne*.

The coding sequences of three *α-Tubulin* genes (*TUA1*, *TUA2*, and *TUA3*; accession nos AF008120, AF008121, and AF008122, respectively) present in goose grass [*Eleusine indica* (L.) Gaertn.] were used to amplify the corresponding genes from tef cDNA using PCR. As tef is allotetraploid, two genomes (A and B) and thus two copies of each gene are expected. Sequencing clones of PCR products revealed no difference between the WT and *kegne* for the ‘*Eragrostis tef α-tubulin 3*’ (*EtTUA3*) gene while no tef orthologue could be amplified for *TUA2.* A point mutation was discovered in *EtTUA1* in one genome of *kegne* (here referred to as copy A) while the other genome (copy B) was unaltered (accession nos KC833037.1, KC833038.1, KC833039.1, KC833040.1, KC833041.1, KC833042.1, KC833043.1, KC833044.1, and KC833045.1). The use of genomic DNA reveals that the *EtTUA1* gene consists of four exons and three introns with a coding sequence of 1353bp (Fig. 5). A cytosine to thymine nucleotide change at position 1596 in the genomic sequence of *kegne* corresponds to the replacement of the polar and uncharged amino acid threonine by the hydrophobic amino acid isoleucine at position 198 (T198I) in the protein sequence.

The TUA1 protein sequence of *Arabidopsis* was searched in the recently published tef genome (Cannarozzi *et al.*, 2014) to identify the scaffolds containing this protein. Scaffold867 and scaffold868 were found to contain the gene copy in which the mutation in TUA1 was located in *kegne* (Supplementary Fig. S7 at *JXB* online). The region upstream and downstream of the *TUA1* gene was investigated for any possible connection to plant architecture (Supplementary Fig. S8). However, none of the eight genes within 100 kbp (50 kbp upstream and 50 kbp downstream) of the *TUA1* gene in the tef genome was known to regulate plant stature.

The multiple sequence alignment of *α-Tubulin* genes of various plants shows that the threonine at position 198 occurs in a conserved region of the alignment and that it is conserved across all plants species analysed (Supplementary Fig. S9 at *JXB* online). Changes at this position have not been tolerated in the seed plants examined, indicating that the amino acid change at position 198 from threonine to an isoleucine could be deleterious to the function of the plant.

### Inheritance of the helical phenotype

The mutation in *kegne* created a recognition site for the *Cla*I restriction enzyme, causing the PCR amplicon for *EtTUA1* of *kegne* to be cleaved while the corresponding amplicon from the WT remained intact due to the absence of the *Cla*I recognition site. Hence, this restriction site was used as a CAPS marker to distinguish between *kegne* and the WT for copy A of *EtTUA1*.

**Fig. 6.**
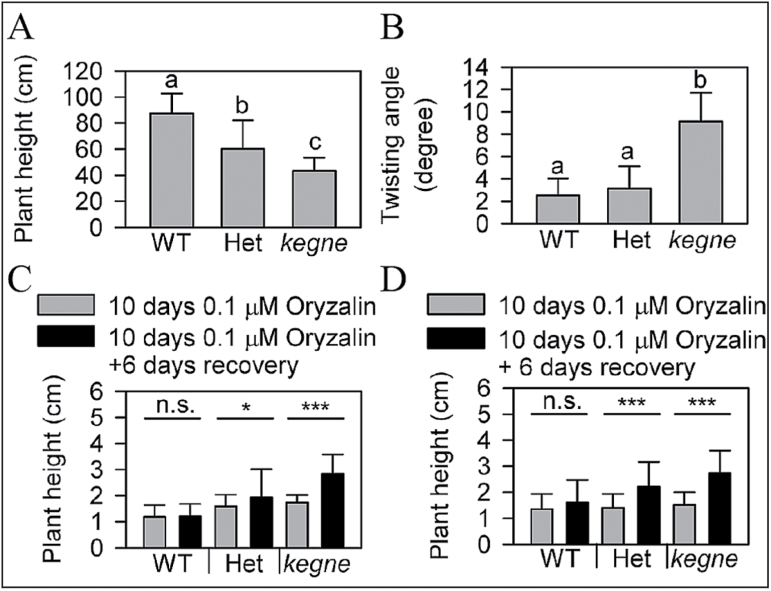
Plant height and angle of twisting in progeny of *kegne* and the *Quncho* cross. (A) Plant height (*n*=121) and (B) angle of twisting (total *n*=114) of F_2_ plants. (C) The height of three classes of F_2_ plants grouped based on the *Cla*I marker after 10 d of oryzalin treatment followed by 6 d of recovery (*n*=93). (D) The height of F_3_ plants derived from a known F_2_ heterozygous line after receiving the same treatment. ***P<0.001; *P<0.05; n.s., not significant after ANOVA. Values with the same letters are not significantly different at *P*<0.05 after Tukey test. Error bars indicate 1 SD.

To investigate the genetic link between the detected mutation and the *kegne* phenotype (i.e. semi-dwarf stature, helical phenotype, and resistance to oryzalin), F_2_ progeny from crosses between *kegne* and several widely cultivated and popular tef cultivars were analysed using the *Cla*I restriction site as a marker. The results showed that reduced plant height and *Cla*I restriction site were linked in all 285 F_2_ progeny studied, indicating that the mutation at this location is tightly linked to the *kegne* phenotype (Table 1). The groups segregated in a ratio of 69:143:73, which is ~1:2:1, suggesting a semi-dominant mutation. Concerning plant height, three distinct groups were observed, with WT plants being the tallest, *kegne* the shortest, and heterozygotes intermediate (Fig. 6A). Regarding the helical phenotype, coleoptiles of F_2_ plants grown in the dark only homozygous for the *Cla*I restriction site showed a strong right-hand twist (Fig. 6B). Although twisting was observed in heterozygous plants, the difference between the WT and heterozygotes was not significant. The F_2_ population and the F_3_ population derived from a heterozygous F_2_ line of the *Quncho*×*kegne* cross were grown on oryzalin for 10 d followed by a 1 week recovery on MS plate without chemical. Obvious recovery was not observed for WT plants, while *kegne* and heterozygous F_2_ plants showed significantly higher growth once they were removed from the chemical (Fig. 6C). Also for F_3_ populations, both the heterozygous and *kegne* lines have significantly higher growth rates compared with the WT (Fig. 6D). Since WT plants were severely affected by the oryzalin treatment (their growth was arrested and the seedlings turned brown), they were unable to recover from the negative effects of oryzalin. However, the growth of both heterozygous and *kegne* plants was significantly improved once they were transferred to medium without the drug.

**Table 1.** The co-segregation test for the three properties of kegne in F_2_ populations involving three crossesAll three properties of *kegne*, namely plant height, helical phenotype, and *Cla*I restriction (given as numbers of plants), were linked. Statistical difference and pairwise comparison was tested with ANOVA.

Crosses (♀×♂)	Phenotype	*Cla*I restriction site			Plant height (cm)	SD	Difference between genotypes	Pairwise comparison
		**+/+**	**+/–**	**–/–**				
*Tsedey*×*kegne*	*Tsedey*	23	0	0	99.61	11.53	*P*=1.46E-31	T versus H: *P*=3.41E-07
	Heterozygous	0	62	0	83.21	12.32		T versus K: *P*=1.24E-25
	*kegne*	0	0	29	49.38	50.86		K versus H: *P*=2.95E-23
*Magna×kegne* ^*a*^	*Magna*	24	0	0	59.04	7.10	*P*=1.31E-31	M versus H: *P*=1.32E-16
	Heterozygous	0	43	0	41.26	5.82		M versus K: *P*=1.38E-24
	*kegne*	0	0	27	30.63	2.65		K versus H: *P*=4.93E-13
*Kay muri*×*kegne*	*Kay muri*	22	0	0	94.04	10.99	*P*=1.65E-24	Km versus H: *P*=6.86E-06
	Heterozygous	0	38	0	79.68	10.06		Km versus K: *P*=1.59E-18
	*kegne*	0	0	17	46.28	7.60		K versus H: *P*=7.15E-18

^*a*^Plant height was measured for progeny of this cross 2 months after sowing unlike for other crosses where the plant height was quantified at physiological maturity.

SD, standard deviation; H, heterozygous; K, *kegne*; Km, *Kay muri*; M, *Magna*; T, *Tsedey*.

### Agronomic performance of *kegne*

*Kegne* plants were evaluated under environmentally controlled conditions in growth rooms in Switzerland. Although panicles of *kegne* were significantly shorter than those of the WT, the differences in total biomass, seed yield, and straw weight were not significant between the two genotypes (Table 2). Interestingly, the 100-seed weight of *kegne* was ~17% heavier than that of the WT. When individual plants were placed at 2cm distance in a tray, *kegne* plants produced higher yield and less overall biomass than the WT, resulting in a higher harvest index (Supplementary Table S1 at *JXB* online).

**Table 2.** Yield and yield-related parameters for WT and *kegne* plants grown under controlled conditions in a growth room (*n*=10; panicle length, WT=44, *kegne*=42)

Parameters	Genotypes		*P*-value
	WT	*Kegne*	
Total biomass per plant (g)	2.03±0.9	1.93±0.34	0.75477
Seed yield per plant (g)	0.77±0.35	0.82±0.15	0.69642
Straw weight per plant (g)	1.26±0.55	1.12±0.22	0.44950
Harvest index (%)	37.8±2.8	42.3±2.6	0.00159
Panicle length (cm)	269±74.1	205±40.5	3.46E-06
100-seed weight (mg)	26±1.5	31±2.0	0.00003

Values are given as mean ± SD. The significance was tested using ANOVA.

## Discussion

In this study, we investigated a novel mutant of tef, a cereal crop which serves as a staple food for ~50 million people in Ethiopia. The mutant, named *kegne*, is the first semi-dwarf tef line and has the potential to overcome lodging, the major yield constraint in tef. *Kegne* is also characterized by helical growth of the first leaf. Such helical growth is a hallmark of mutations affecting the cytoskeleton. Indeed, microtubule drug assays, microtubule immunolabelling, co-segregation of the semi-dwarfism, and the helical phenotype with a polymorphism in the *EtTUA1* gene strongly suggest that the *kegne* phenotype is due to a single base mutation in this gene.

Microtubules play an important role in cell elongation (Wang and Li, 2008). The helical leaves (Fig. 1B) and the twisting cell files in dark-grown coleoptiles (Fig. 1D) of *kegne* were similar to those described for other species affected in microtubule organization. In *Arabidopsis*, mutations in microtubule-associated genes such as *TORTIFOLIA 1* (*TOR1*) resulted in helical growth of roots and aerial organs (Buschmann *et al.*, 2004). Mutations in *α-* or *β-Tubulin* genes, such as the *lefty Arabidopsis* mutants, induce a similar phenotype (Thitamadee *et al.*, 2002; Ishida *et al.*, 2007*a*). In addition to their helical growth, microtubule-associated mutants have been reported to be more sensitive to propyzamide and taxol, microtubule-depolymerizing and -stabilizing drugs, respectively (Furutani *et al.*, 2000; Buschmann *et al.*, 2004; Shoji *et al.*, 2004). The most striking effect of propyzamide was observed in WT plants grown under both light and dark conditions. Three-day-old WT shoots on propyzamide media were converted to the typical helical phenotype exhibited in *kegne* plants (Fig. 3A). This strongly suggests that this *kegne* phenotype was due to a mutation in a microtubule-related gene. In addition, *kegne* plants were found to be resistant to oryzalin (Figs 3, 6), a drug known to disrupt microtubules by binding specifically to α-tubulins (Murthy *et al.*, 1994; Morrissette *et al.*, 2004). Investigations of F_2_ progeny from crosses between *kegne* and the WT after treatment with 0.1 μM oryzalin reveals three groups which correspond to homozygous WT, heterozygous, and homozygous *kegne* (Fig. 6). Similar results were obtained in progeny of crosses between *kegne* and other tef cultivars. These genotypes correspond to lines with extreme, medium, and low sensitivity to oryzalin, respectively. Similar to *kegne*, mutations in *α-Tubulin* genes of *E. indica* confer resistance to oryzalin and other dintroaniline (Vaughn *et al.*, 1987; Yamamoto and Baird, 1999; Yamamoto *et al.*, 1998). This supports the assumption that the phenotype of *kegne* is linked to a mutation in the *α-Tubulin* gene.

Additional evidence for this hypothesis is the phenotypic similarity of *kegne* to other *α-tubulin* mutants. For instance, a non-synonymous amino acid substitution in an *α-Tubulin* gene product led to semi-dwarfism in the *tid1* mutant of rice, which also shows helical growth of leaves (Sunohara *et al.*, 2009). In the case of the *tid1* mutant, heterozygous plants clearly resemble homozygous *kegne* mutant plants, whereas homozygous *tid1* mutants are severely dwarf. This severe dwarfing of the homozygous *tid1* mutant is due to the diploid nature of rice, whereas the *kegne* mutation is present in only one genome copy of the allotetraploid tef species. The other genome copy has an unaffected orthologous gene, indicating that each genome has an additive effect on plant height in tef. This additive contribution of the two tef genomes to plant height and helical growth could also be explained from the heterozygous F_2_ plants which attained intermediate levels of plant height as shown in Fig. 3.

It is suggested that a single base pair change in only one copy of the *EtTUA1* gene of *kegne*, that converts the amino acid threonine (polar, uncharged) to the amino acid isoleucine (hydrophobic) at position 198 (T198I; Fig. 5; Supplementary Fig. S9 at *JXB* online), causes the *kegne* phenotype. Referring to the best studied structure of the pig α-tubulin, the described mutation is sited towards the central region of the protein, just before β-sheet six (Nogales *et al.*, 1999; Lowe *et al.*, 2001). Investigations in other systems showed that mutations in *α-Tubulin* genes can lead to right- or left-handed helical growth of aerial organs and roots, depending on the position of the mutation (Ishida *et al.*, 2007*a*). In most cases, these mutations were proposed to be positioned in regions involved in longitudinal contacts between α-/β-tubulin heterodimers, or in regions important for lateral contacts of protofilaments. Nevertheless, mutations outside these regions can also induce helical growth. For example, the non-synonymous amino acid substitution D205N, just after β-sheet six, induced left-handed helical growth, and a non-synonymous substitution at position 268 in the neighbouring β-sheet seven induced right-handed growth in *Arabidopsis* (Ishida *et al.*, 2007*a*). Hence, the amino acid substitution T198I in *kegne* could be responsible for the typical helical phenotype of the mutant, since it is also placed just before one of the β-sheets in this region of the protein. The same region around the mentioned β-sheets in the core of the α-tubulin protein might also mediate oryzalin resistance. In the model organism *Toxoplasma*, oryzalin resistance was conferred by amino acid alteration in β-sheet one, β-sheet four, and just before β-sheet five (Morrissette *et al.*, 2004).

The resistance of *kegne* to oryzalin (Fig. 3C, E) might also confer resistance to other non-selective dintroaniline-based herbicides. This could have huge importance especially in controlling graminaceous weeds from tef fields. Resistance to oryzalin and other dintroaniline herbicides due to *α-tubulin* mutations were reported for monocots such as *E. indica* and *S. viridis* (Yamamoto *et al.*, 1998; Delye *et al.*, 2004). However, the maximum tolerance level of 0.1 μM oryzalin obtained for *kegne* is far less than 1 μM oryzalin reported for *E. indica* (Vaughn *et al.*, 1987). It is therefore important to determine the tolerance level of *kegne* in the field to several non-selective herbicides and also the efficacy of these herbicide concentrations against major grass weeds in tef cultivation.

Twisting phenotypes in plants with mutations in microtubule-related genes are often coupled with microtubule arrays of a specific handedness in the epidermis. However, the direction of twisting in the epidermis is opposite to the orientation of microtubule arrays. This was confirmed earlier from studies on *Arabidopsis* in which the right-handed twisting mutants such as *spr1* and *tor1* attained left-handed microtubule arrays (Furutani *et al.*, 2000; Buschmann *et al.*, 2004). In addition, the left-handed twisting mutants such as *lefty* and *mor1* had right-handed microtubule arrays (Whittington *et al.*, 2001; Thitamadee *et al.*, 2002). As cortical microtubules guide the deposition of cellulose microfibrils in cell walls and cell expansion is usually perpendicular to the net orientation of cellulose microfibrils (Baskin, 2005), microtubule arrays of a specific handedness were suggested to induce helical growth and thereby twisting plant phenotypes. Accordingly, helical growth in *Arabidopsis tor1* or *tor2* mutants is preceded by a shift of microtubule orientation into a specific handedness, which suggests that changes in microtubule organization in twisting mutants are a cause rather than a result of helical growth (Buschmann *et al*., 2004, 2009). Based on these studies and the present findings, it is concluded that the twisting phenotype in the *kegne* mutant is a consequence of the dominant left-handed microtubule arrays.

The present findings show that *kegne* is not impaired in either the biosynthesis or signalling of plant hormones such as BR and GA. However, paclobutrazol, a GA biosynthesis inhibitor, which significantly reduced the height of WT tef in the dark, had little effect on *kegne* shoots grown in the dark and roots grown in the light (Supplementary Fig. S3 at *JXB* online). This might happen if *kegne* is already impaired in cell elongation due to features related to low dynamic microtubules, hence the effect of reduced GA levels caused by paclobutrazol application could be masked. As a result, *kegne* might be resistant to certain concentrations of paclobutrazol.

The favourable characteristics of *kegne* under controlled conditions prompted the assessment of *kegne* and respective crosses to popular tef cultivars such as *Quncho* (Assefa *et al.*, 2011) under field conditions in Ethiopia. *Quncho* was developed from the cross between the improved varieties *Magna*, a variety with consumer-preferred white grain colour but low productivity, and *Dukem*, a high yielding variety but with low market price due to the pale white grain colour. *Quncho* harbours both desirable traits, namely the white seed colour and the high yield. As a result, this variety has received high acceptance by tef farmers. However, since *Quncho* is tall and prone to lodging, the introgression of *kegne* will improve the standing ability of the former and as a consequence will further increase the productivity of tef. As a general rule, after hybridization, progenies need to be grown for at least eight generations in order to achieve a level of homozygosity acceptable to the authorities that give permission for the release of a crop as a new cultivar. The variety selection mechanism involves not only researchers but also farmers in a system known as Farmer Participatory Research (Belay *et al.*, 2006, 2008). Once the new varieties are approved for release, seeds are multiplied by seed multipliers involving public and private seed companies as well as seed grower farmers’ associations. It is firmly believed that the semi-dwarf and lodging-tolerant lines derived from crosses to *kegne* will boost productivity of tef in farmers’ fields since lodging, the major yield-limiting factor in tef cultivation, will be tackled.

In summary, *kegne* is the first semi-dwarf and lodging-tolerant tef line, characterized by helical growth which is probably caused by a mutation in the *α-Tubulin* gene *EtTUA1*. Although complementation of *kegne* with the WT allele could not be carried out at the present time due to the lack of a stable transformation method for tef, the findings from microtubule drug assays, immunolabelling, and sequencing strongly suggest that the *kegne* phenotype, namely the semi-dwarf stature, helical growth, and oryzalin resistance, are due to a single base pair mutation in the *α-Tubulin* gene that replaces the polar and uncharged amino acid threonine with the hydrophobic amino acid isoleucine at position 198 of EtTUA1. The unique mutation in this highly conserved region of the *α-Tubulin 1* gene among many plant species examined suggests that it is the cause of the drastic changes in the plant architecture of the *kegne* mutant. The present work highlights the advantages and opportunities associated with an indigenous crop. Major yield gains will reach subsistence farmers efficiently through well-established seed multiplication and distribution channels.

## Supplementary data

Supplementary data are available at *JXB* online.

Supplementary methods.

Figure S1. Height differences between *kegne* and the wild type.

Figure S2. Effect of different levels of gibberellic acid on the shoot and root length of wild-type and *kegne* plants grown *in vitro* for 3 d.

Figure S3. Effect of different levels of paclobutazol on the shoot and root length of wild-type and *kegne* plants grown *in vitro* for 3 d.

Figure S4. Effect of different levels of brassinolide on the shoot and root length of wild-type and *kegne* plants grown *in vitro* for 3 d.

Figure S5. Effect of different levels of brassinozole220 on the shoot and root length of wild-type and *kegne* plants grown *in vitro* for 3 d.

Figure S6. Effect of different propyzamide concentrations on the shoot and root length of wild-type and *kegne* plants grown *in vitro* for 3 d.

Figure S7. Phylogenetic tree of α-tubulin protein sequences from diverse plant species.

Figure S8. Comparison of orthologous syntenic genomic regions between tef and rice.

Figure S9. Alignment of *α-Tubulin* genes in plants.

Table S1. Yield and yield components of 312 wild-type and *kegne* plants grown in a tray of 0.128 m^2^ under equal day conditions.

Supplementary Data

## Abbreviations:

BL: brassinolide
BR: brassinosteroid
BSA: Bovine Serum Albumin
CAPS: Cleaved Amplified Polymorphic Sequence
CDS: Coding sequence
CSA: Central Statistical Agency (Ethiopia)
DMSO: dimethylsufoxide
EMS: ethyl methanesulphonate
FITC: Fluorescein isothiocyanate
GA: gibberellic acid
IAA: indole-3-acetic acid
MAP: microtubule-associated protein
MS: Murashige and Skoog
MTSB: microtubule-stabilizing buffer
NPA: *n*-1-naphthylphthalamic acid
PBS: phosphate-buffered saline
SEM: scanning electron microscope.
