# Supplementary material for: Semi-dwarfism and lodging tolerance in tef (Eragrostis tef) is linked to a mutation in the α-Tubulin 1 gene
Source: J Exp Bot. 2014 Nov 15;66(3):933–44. doi: 10.1093/jxb/eru452 (PMC4321551; doi:10.1093/jxb/eru452)
Supplement: Supplementary Data [file supp_66_3_933__index.html]

Semi-dwarfism and lodging tolerance in tef (Eragrostis tef) is linked to a mutation in the α-Tubulin 1 gene — Semi-dwarfism and lodging tolerance in tef (Eragrostis tef) is linked to a mutation in the α-Tubulin 1 gene — Supplementary Data 

# Semi-dwarfism and lodging tolerance in tef (*Eragrostis tef*) is linked to a mutation in the *α-Tubulin 1* gene

## Supplementary Data

Data files

**Files in this Data Supplement:**

- Supplementary Data - Supplementary Data
