# Supplementary material for: Semi-dwarfism and lodging tolerance in tef (Eragrostis tef) is linked to a mutation in the α-Tubulin 1 gene
Source: J Exp Bot. 2014 Nov 15;66(3):933–44. doi: 10.1093/jxb/eru452 (PMC4321551; doi:10.1093/jxb/eru452)
Supplement: Supplementary Data [file supp_eru452_jexbot130856_file002.pdf]

Semi-dwarfism and lodging tolerance in tef (*Eragrostis tef*) is linked to a mutation in the *α-Tubulin 1* gene. Moritz Jöst, Korinna Esfeld, Agata Burian, Gina Cannarozzi, Solomon Chanyalew, Cris Kuhlemeier, Kebebew Assefa and Zerihun Tadele

SUPPLEMENTARY DATA

Supplementary Methods

Sequence retrieval and analysis: *Arabidopsis* TUA1 sequences were downloaded from TAIR (TAIR): TUA1 (AT1G64740.1), TUA2 (AT1G50010.1), TUA3 (AT5G19770.1), TUA4 (AT1G04820.1), TUA5 (AT5G19780.1), and TUA6 (AT4G14960.1). The following databases were obtained from Phytozome (Goodstein *et al.*, 2012): *Arabidopsis thaliana* (Phytozome, v. 79) (Lamesch *et al.*, 2012), *Sorghum bicolor* (Phytozome, v. 79) (Paterson *et al.*, 2009), *Zea mays* (Phytozome, v. 181) (Schnable *et al.*, 2009), *Setaria italica* (Phytozome, v. 164) (Bennetzen *et al.*, 2012) and *Brachypodium distachyon* (Phytozome, v. 192) (Vogel *et al.*, 2010). The *Oryza sativa* genome was retrieved from IRGSP (version 1.0, 2011-12-05) (Kawahara *et al.*, 2013; Sakai *et al.*, 2013). The *Eragrostis tef* genome was retrieved from the Tef Improvement Project (Cannarozzi *et al.*, 2014).

The TUA1 protein sequence was used to search the protein sequences of each of the genomes using blastp with E-value <= 10^{-15} (Altschul *et al.*, 1990). This resulted in *α*-, *β*- and *γ-Tubulin* genes. Only the *α*- and one *β*- (as outgroup) *Tubulin* genes were retained and redundant sequences were pruned. Sequences were kept for *Setaria italica* (Si029822m, Si035680m, Si035654m, Si035662m, Si035763m), *Zea mays* (GRMZM2G083243_T01, GRMZM2G099167_T01, GRMZM2G152466_T03, GRMZM2G051782_T01, GRMZM2G153292_T01, AC195340.3_FGT001), *Oryza sativa* (Os07t0574800-01, Os03t0219300-01, Os03t0726100-01, Os11t0247300-01), *Bracypodium distachyon* (Bradi1g23770.1, Bradi1g69670.1, Bradi1g10150.1, Bradi1g07160.1), *Eragrostis tef* (Et_s942-0.37-1, Et_s867-1.8-1, Et_s2744-0.8-1, Et_s7282-0.60-1, Et_s868-1.12-1, Et_s2676-0.58-1, Et_s2885-0.20-1, Et_s3288-1.13-1), and *Sorghum bicolor* (Sb01g042650.1, Sb01g009560.1, Sb01g006650.1, Sb01g009570.1). The following were obtained from NCBI: *Eleusine indica* (AAC05719, O22347, AAC05718), *Triticum aestivum* (ABD92942.1, ABD92937), *Prunus mume* (XP_008240533.1), *Malus domestica* (XP_008349406.1, XP_008372961.1), *Populus tremuloides* (ABR67425.1, AAO63773.2), *Populus trichocarpa* (XP_002303998.1, XP_002298794.2, XP_002324047.2), *Ricinus communis* (XP_002532068.1, XP_002525416.1, XP_002510010.1), *Capsella rubella*

(XP_006286664.1, EOA40391.1), *Glycine max* (NP_001242878.1, XP_003522731), *Phoenix dactylifera* (XP_008813089, XP_008804464), *Eucalyptus grandis* (KCW63554.1), *Coffea canephora* (CDP14299.1), *Gossypium_hirsutum* (ABO47735.1), *Theobroma cacao* (XP_007042689.1), *Salix arbutifolia* (AGG37288.1) and *Cicer arietinum* (XP_004496862.1). In addition the *Eragrostis tef* A and B copies (Tua1_copyA_WT37, Tua1_copyB_WT37), the *kegne* mutant (Tua1_copyA_3774-13) and the *Oryza_sativa tid1* mutant (BAH85357.1) were included. *β-Tubulin* from *Arabidopsis thaliana* (AT2G29550.1) was used as an outgroup.

Alignment: The protein sequences were aligned using Mafft v6.903b (Katoh *et al.*, 2005; Larkin *et al.*, 2007; Thompson *et al.*, 1994) with the default parameters. If one sequence had an insertion of greater than 20 bases pairs not present in any other sequences, this insertion was trimmed from the sequence.

Tree: PhyML (Guindon *et al.*, 2005) was used to obtain a maximum likelihood tree using the default parameters. Branch support was inferred using the Shimodaira–Hasegawa-like (SH) aLRT provided by PhyML. The phylogenetic tree was visualized using FigTree v1.3.1 (Rambaut).

Analysis: The CoGeBlast tool of the CoGe website was used to find the scaffolds in *Eragrostis tef* (coge id: id38364) and *Oryza sativa japonica* (coge id: id3) closest to the *kegne* mutant. These genomic segments were analyzed with GEvo at the same website. The analysis can be reproduced at: http://www.genomevolution.org/r/dljt.

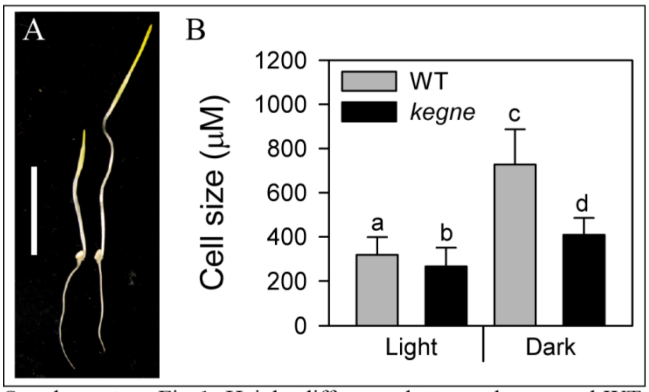

Supplementary Fig 1. Height difference between kegne and WT. The coleoptiles of kegne were shorter than the WT under both light and dark conditions. (A) kegne (left) and WT (right) seedlings grown for three days in the dark. Scale bar 1 cm. (B) Length of coleoptile cells of the two genotypes grown for four days in the light or three days in the dark. Values with the same letters are not significantly different at the $p < 0.05$ level after Tukey-test. Error bars indicate one standard deviation (dark: N = 48, light: N = 96).

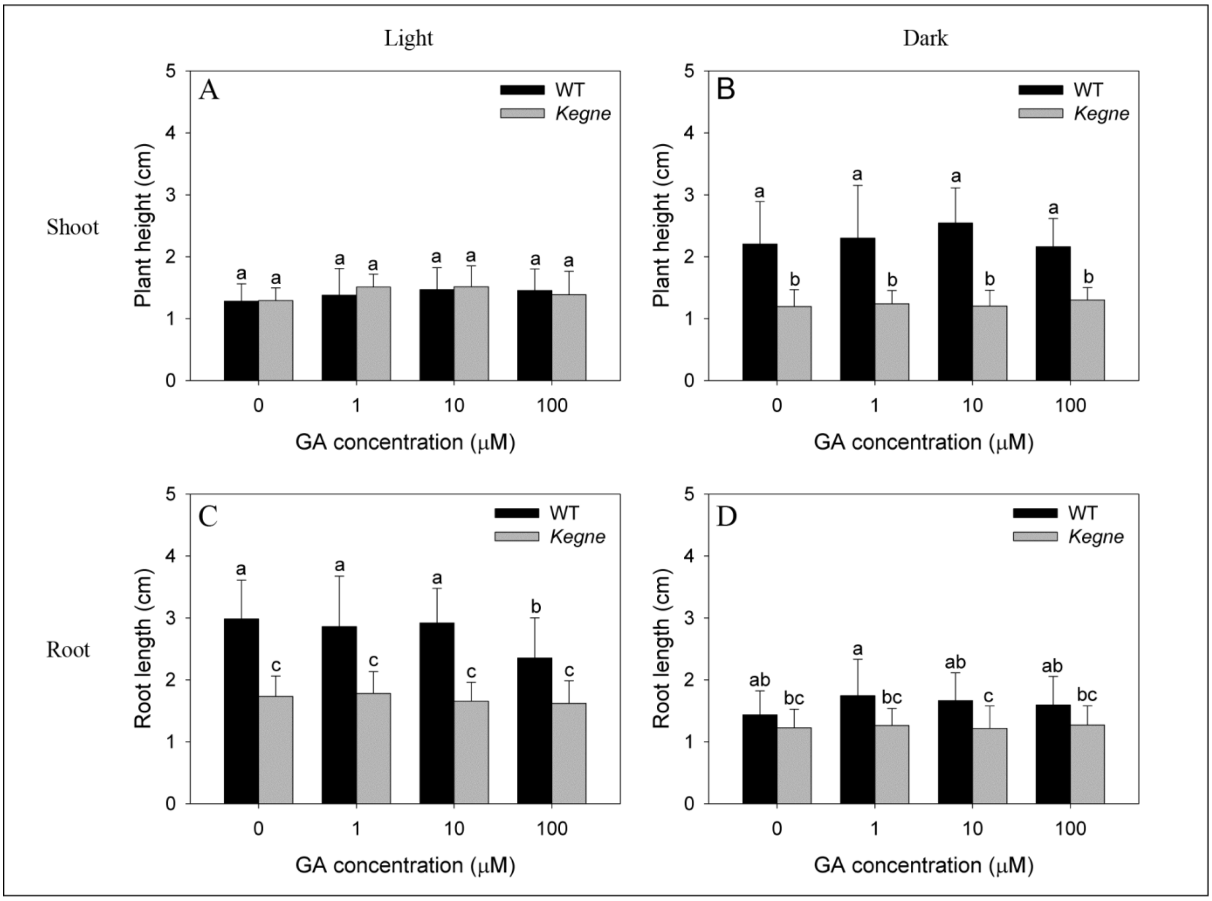

Supplementary Fig 2. Effect of different levels of gibberrelic acid (GA) on the shoot and root length of WT and kegne plants grown in vitro for three days. Plant height for light (A) and dark (B); and root length for light (C) and dark (D) grown plants. A minimum of 13 plants per treatment were measured. Values with the same letters are not significantly different at the $p < 0.05$ level using a Tukey-test. Error bars indicate one standard deviation.

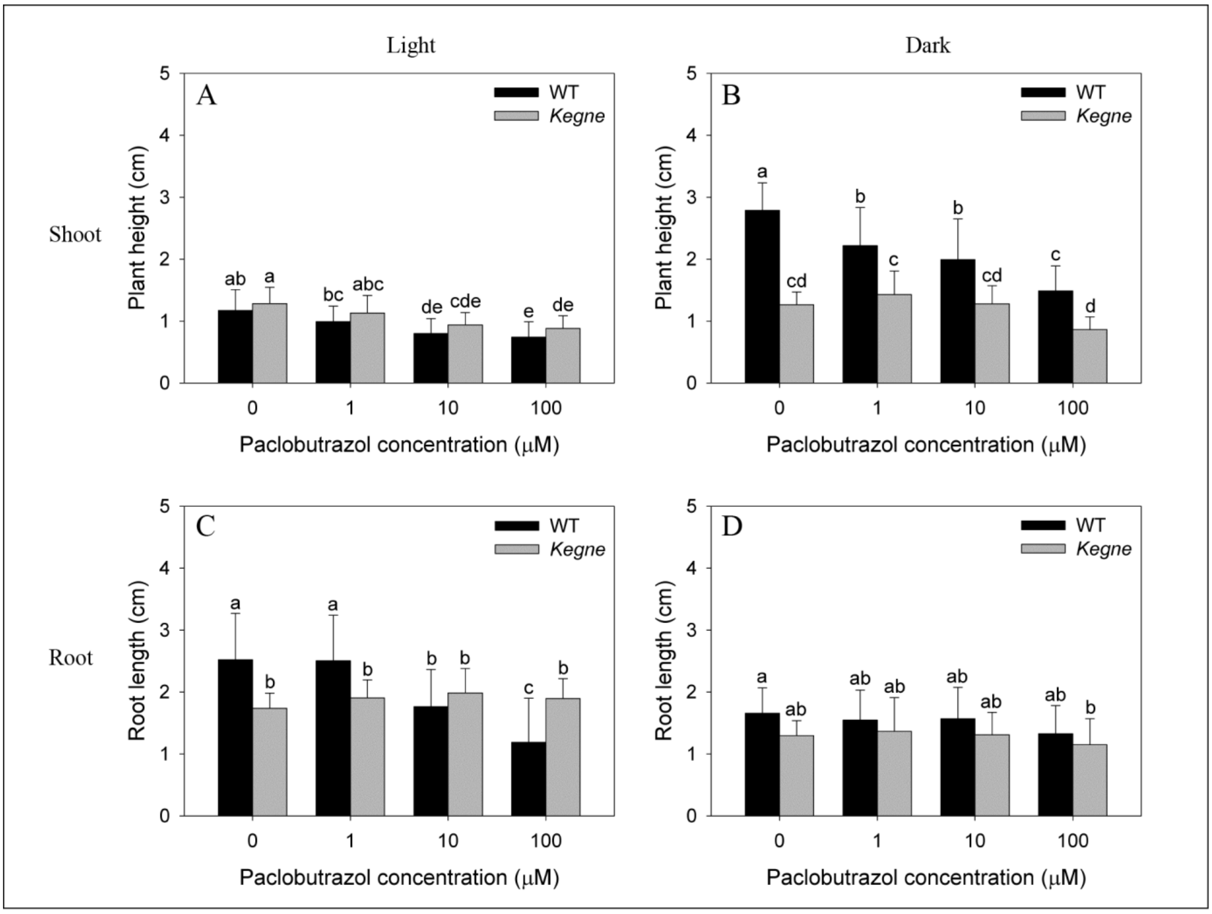

Supplementary Fig 3. Effect of different levels of paclobutrazol on the shoot and root length of WT and kegne plants grown in vitro for three days. Plant height for light (A) and dark (B); and root length for light (C) and dark (D) grown plants. A minimum of 11 plants per treatment were measured. Values with the same letters are not significantly different at the
$p < 0.05$ level using the Tukey-test. Error bars indicate one standard deviation.

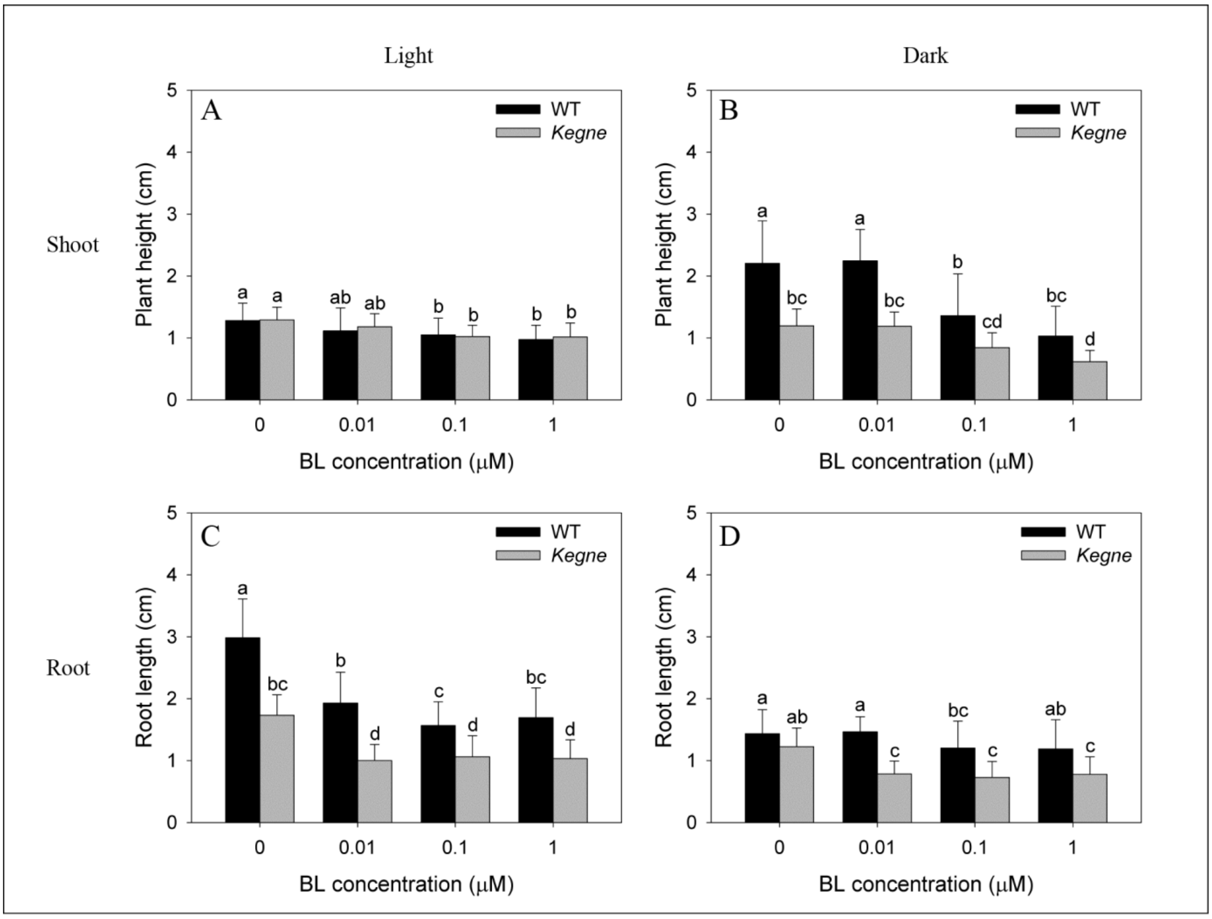

Supplementary Fig 4. Effect of different levels of brassinolide (BL) on the shoot and root length of WT and kegne plants grown in vitro for three days. Plant height for light (A) and dark (B); and root length for light (C) and dark (D) grown plants. A minimum of 14 plants per treatment were measured. Values with the same letters are not significantly different at the $p < 0.05$ level after Tukey-test. Error bars indicate one standard deviation.

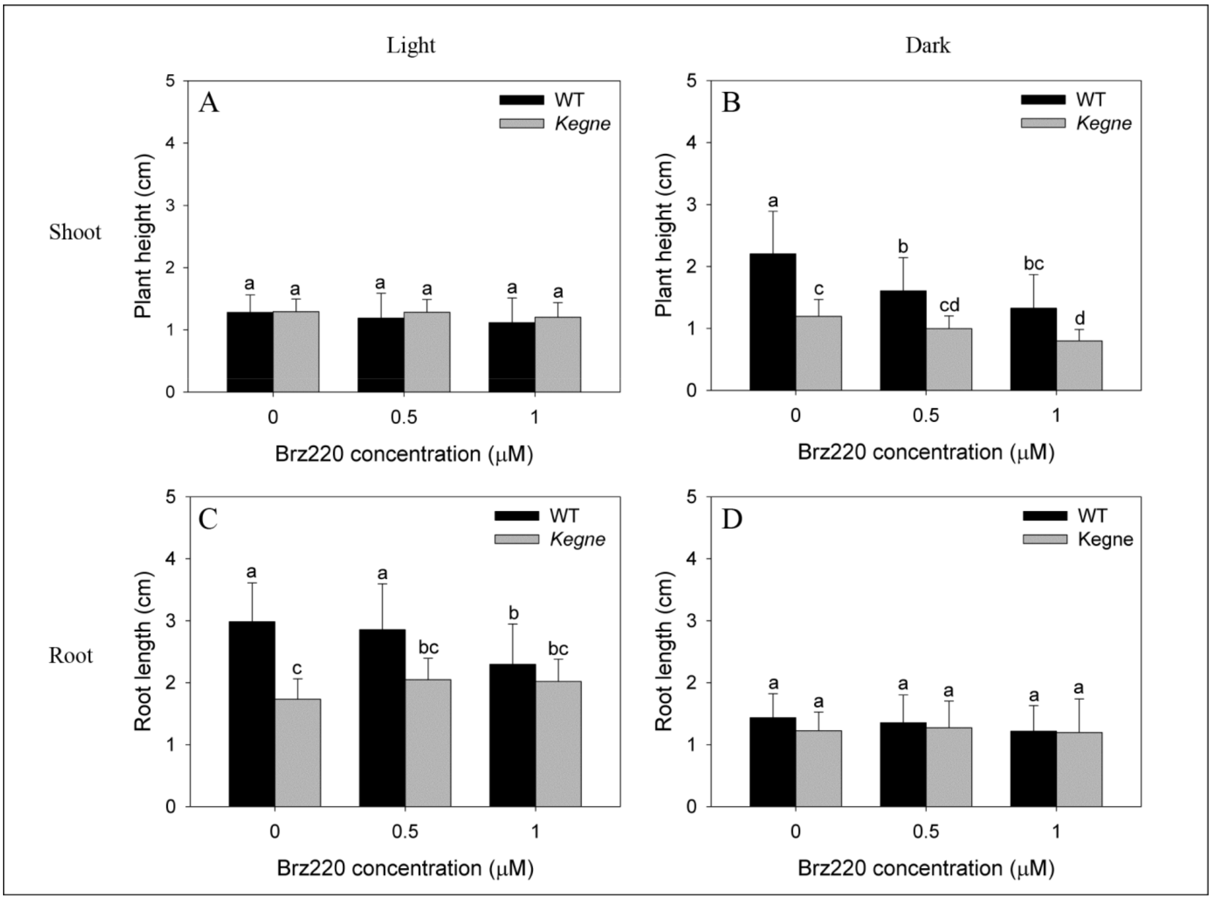

Supplementary Fig 5. Effect of different levels of brassinozole220 (Brz220) on the shoot and root length of WT and kegne plants grown in vitro for three days. Plant height for light (A) and dark (B); and root length for light (C) and dark (D) grown plants. A minimum of 14 plants per treatment were measured. Values with the same letters are not significantly different at the $p < 0.05$ level after Tukey-test. Error bars indicate one standard deviation.

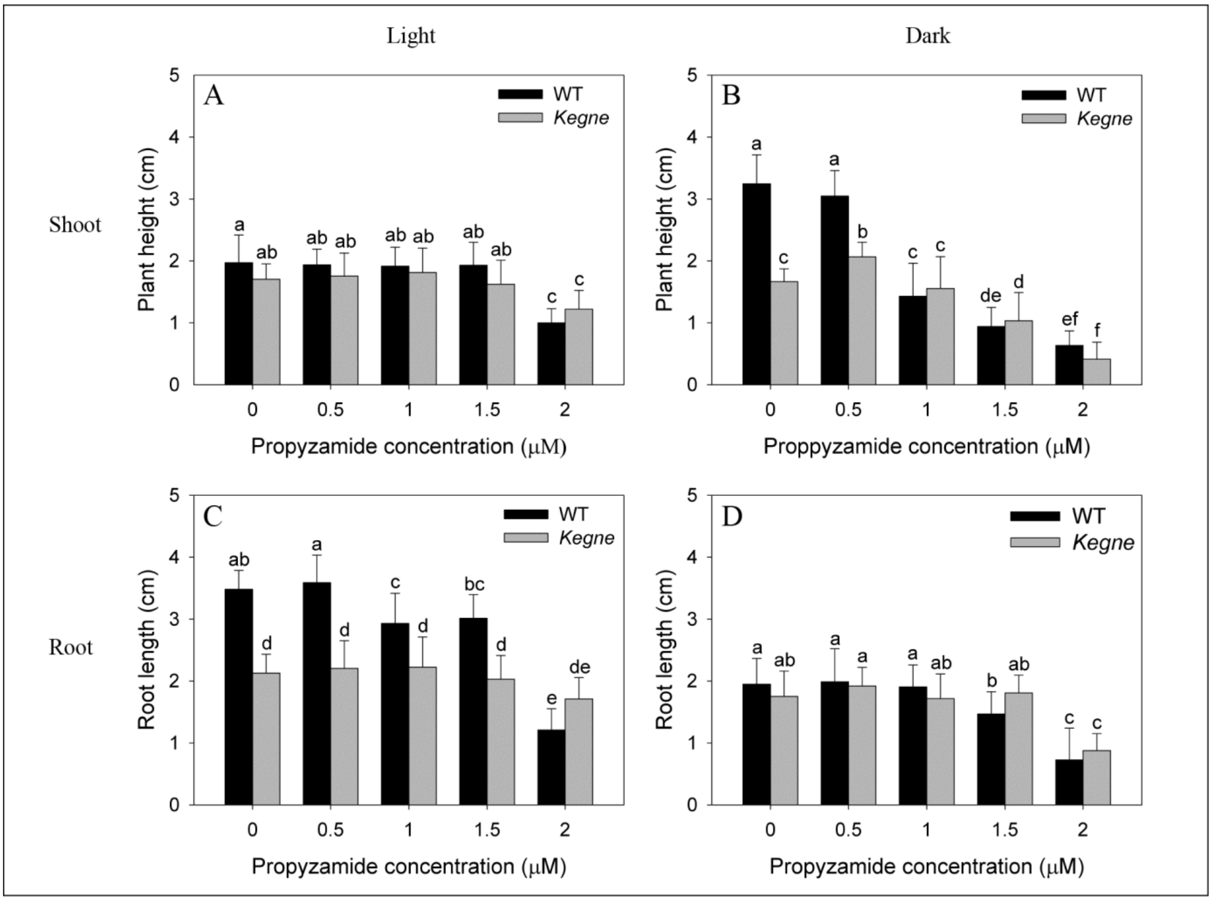

Supplementary Fig 6. Effect of different propyzamide concentrations on shoot and root length of WT and kegne plants grown in vitro for three days. Plant height for light (A) and dark (B); and root length for light (C) and dark (D) grown plants. A minimum of 22 plants were measured for each treatment. Values with the same letters are not significantly different at the $p < 0.05$ level after Tukey-test. Error bars indicate one standard deviation.

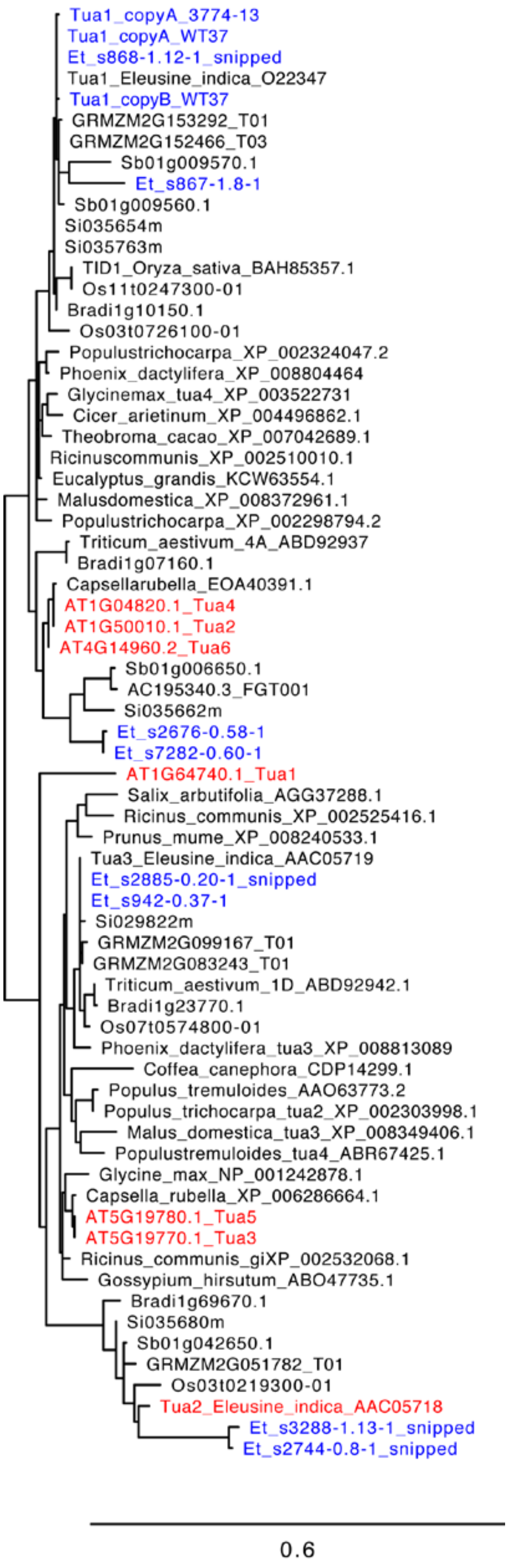

Supplementary Fig. 7. Phylogenetic tree of α-Tubulin protein sequences from diverse plant species. The coding sequences for various species were obtained from Phytozome, NCBI, the tef genome (http://www.tef-research.org) and IRGSP as described in the methods. Tef sequences are shown in blue while *Arabidopsis* sequences are shown in red. The maximum likelihood tree was inferred using PhyML with the default parameters. The scale bar reflects evolutionary distance, measured in units of substitution per site. Branch support was inferred using the Shimodaira-Hasegawa-like (SH) aLRT provided by PhyML. The tree was rooted with a β-Tubulin from *Arabidopsis* (not shown).

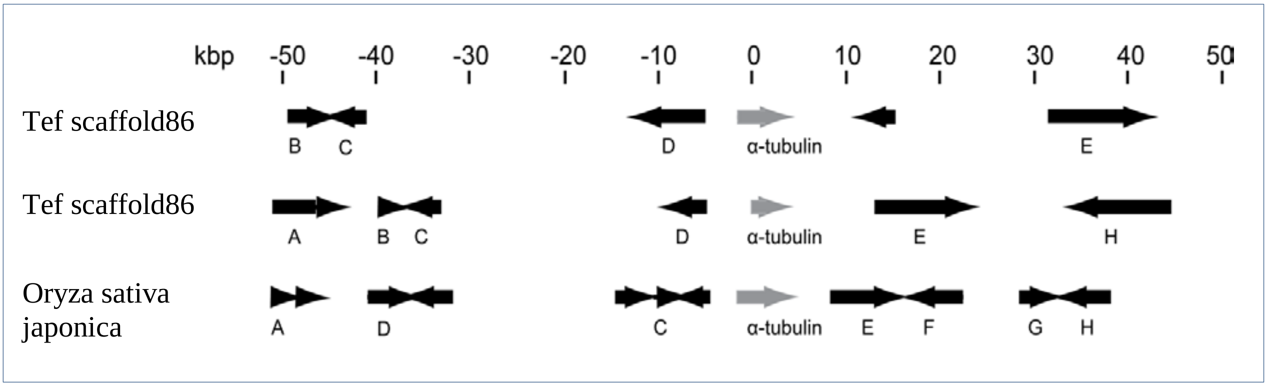

Supplementary Fig. 8. Comparison of orthologous syntenic genomic regions between tef and rice. The genes in the tef genome (Cannarozzi *et al.*, 2014) closest to the TUA1 gene were identified on scaffold867 and scaffold868. This region on these two scaffolds ~~is~~ was compared to the orthologous region on the rice genome. All genes within 50 kbp upstream and downstream were investigated. None of the eight genes in the region had any reported effect on plant stature. The eight genes in close proximity to the TUA1 gene in the tef and rice genomes are briefly described below.

Gene A [FIP1(FIN219-interacting protein; rice: Os03g51520.2; At1g78370] is regulated by light whereby the overexpression of FIP1 in *Arabidopsis* plants resulted in a hyposensitive hypocotyl phenotype under continuous far-red light and delayed flowering under long-day conditions (Chen *et al.*, 2007).

Gene B [Lariat debranching enzyme; Arabidopsis: At4g31770] has been named as AtDBR1 (for Arabidopsis thaliana Debranching enzyme 1) and is responsible for early development during the embryo stage (Wang *et al.*, 2004). *Arabidopsis* mutants altered in this gene possess defective embryos starting from the early globular stage of embryogenesis.

Gene C [Basic helix-loop-helix (bHLH) family protein; rice: Os03g51580.1; AT2G22770.1]. bHLH proteins are a superfamily of transcription factors that are important in regulatory components in diverse biological processes especially in the photoreceptor signaling network (Toledo-Ortiz *et al.*, 2003).

Gene D [WD repeat containing protein 5, putative expressed; rice: Os03g51550.2]. Repeated WD motifs act as a site for protein-protein interaction, and proteins containing WD repeats (WDRs) are known to serve as platforms for the assembly of protein complexes or mediators of transient interplay among other proteins (van Nocker and Ludwig, 2003). These proteins act as scaffolding molecules assisting proper activity of other proteins.

Gene E [Inositol 1,3,4-triphosphate 5/6 kinase, putative; rice: Os03g51610; Arabidopsis: At4g33770.1] is essential for drought and salt stresses responses in rice (Du *et al.*, 2011). Both mutants defective in the gene and those which overexpress it are hypersensitive to drought and salt.

Gene F [DUF221 domain containing protein, expressed (rice: Os03g51620.1; Arabidopsis: At4g22120)]. It encodes an integral membrane protein with eight or more predicted transmembrane helices. The protein is an ion channel that can be activated by hyperosmotic shock (Hou *et al.*, 2014).

Gene G [uncharacterized protein (rice: Os03g51640.1)]

Gene H [Sulfite exporter Tau E/Saf E family proten (rice: Os03g51650.1; Arabidopsis: At2636630.1)]

```
AT1G64740.1_Tua SKLGFTIYPSPQVSTAVVEPYNSVLSTHSLLEHTDVVVLLDNEAIYDICRRSLDIERPTY
AT5G19770.1_Tua SKLGFTIYPSPQVSTAVVEPYNSVLSTHSLLEHTDVAVLLDNEAIYDICRRSLDIERPTY
AT5G19780.1_Tua SKLGFTIYPSPQVSTAVVEPYNSVLSTHSLLEHTDVAVLLDNEAIYDICRRSLDIERPTY
AT1G50010.1_Tua SKLGFTVYPSPQVSTSVVEPYNSVLSTHSLLEHTDVSILLDNEAIYDICRRSLSIERPTY
AT1G04820.1_Tua SKLGFTVYPSPQVSTSVVEPYNSVLSTHSLLEHTDVSILLDNEAIYDICRRSLSIERPTY
AT4G14960.2_Tua SKLGFTVYPSPQVSTSVVEPYNSVLSTHSLLEHTDVSILLDNEAIYDICRRSLNIERPTY
Si029822m       SKLGFTIYPSPQVSTAVVEPYNSVLSTHSLLEHTDVAVLLDNEAIYDICRRSLDIERPTY
Si035680m       SKLGFTIYPSPQISTAVVEPYNSVLSTHSLIEHTDVVVLLDNEAIYDICKRSLDIERPTY
Si035662m       SKLGFTVYPSPQVSTSVVEPYNSVLSTHSLLEHTDVSILLDNEAIYDICRRSLDIERPNY
Si035763m       SKLGFTVYPSPQVSTSVVEPYNSVLSTHSLLEHTDVAVLLDNEAIYDICRRSLDIERPTY
GRMZM2G083243_T SKLGFTIYPSPQVSTAVVEPYNSVLSTHSLLEHTDVAVLLDNEAIYDICRRSLDIERPTY
GRMZM2G099167_T SKLGFTIYPSPQVSTAVVEPYNSVLSTHSLLEHTDVAVLLDNEAIYDICRRSLDIERPTY
GRMZM2G051782_T SKLGFTIYPSPQISTAVVEPYNSVLSTHSLIEHTDVVVLLDNEAIYDICKRSLDIERPTY
GRMZM2G153292_T SKLGFTVYPSPQVSTSVVEPYNSVLSTHSLLEHTDVAILLDNEAIYDICRRSLDIERPTY
AC195340.3_FGT0 SKLGFTVYPSPQVSTSVVEPYNSVLSTHSLLEHTDVSILLDNEAIYDICRRSLDIERPNY
Os07t0574800-01 SKLGFTIYPSPQVSTAVVEPYNSVLSTHSLLEHTDVAVLLDNEAIYDICRRSLDIERPTY
Os03t0219300-01 SKLGFTIYPSPQISTAVVEPYNSVLSTHSLIEHTDVVVLLDNEAIYDICKRSLDIERPTY
Os03t0726100-01 SKLGFTVYPSPQVSTSVVEPYNSVLSTHSLLEHTDVAVLLDNEAIYDICRRSLDIERPTY
Os11t0247300-01 SKLGFTVYPSPQVSTSVVEPYNSVLSTHSLLEHTDVAVLLDNEAIYDICRRSLDIERPTY
Bradi1g23770.1  SKLGFTIYPSPQVSTAVVEPYNSVLSTHSLLEHTDVAVLLDNEAIYDICRRSLDIERPTY
Bradi1g69670.1  SKLGFTIYPSPQISTAVVEPYNSVLSTHSLIEHTDVVVLLDNEAIYDICKRSLDIERPTY
Bradi1g10150.1  SKLGFTVYPSPQVSTSVVEPYNSVLSTHSLLEHTDVAVLLDNEAIYDICRRSLDIERPTY
Bradi1g07160.1  SKLGFTVYPSPQVSTSVVEPYNSVLSTHSLLEHTDVSILLDNEAIYDICRRSLDIERPTY
Et_s867-1.8-1   SKLGFTVYPSPQVSTSVVEPYNSVLSTHSLLEHTDVAVLLDNEAIYDICRRSLDIERPTY
Et_s868-1.12-1_ SKLGFTVYPSPQVSTSVVEPYNSVLSTHSLLEHTDVAVLLDNEAIYDICRRSLDIERPTY
Sb01g042650.1   SKLGFTIYPSPQISTAVVEPYNSVLSTHSLIEHTDVVVLLDNEAIYDICKRSLDIERPTY
Sb01g009560.1   SKLGFTVYPSPQVSTSVVEPYNSVLSTHSLLEHTDVAVLLDNEAIYDICRRSLDIERPTY
Sb01g006650.1   SKLGFTVYPSPQVSTSVVEPYNSVLSTHSLLEHTDVSILLDNEAIYDICRRSLDIERPNY
Sb01g009570.1   SKLGFTVYPSPQVSTSVVEPYNSVLSTHSLLEHTDVAVLLDNEAIYDICRRSLDIERPTY
Tua1_copyA_WT37 SKLGFTVYPSPQVSTSVVEPYNSVLSTHSLLEHTDVAVLLDNEAIYDICRRSLDIERPTY
Tua1_copyB_WT37 SKLGFTVYPSPQVSTSVVEPYNSVLSTHSLLEHTDVAVLLDNEAIYDICRRSLDIERPTY
Tua1_copyAkegne SKLGFTVYPSPQVSTSVVEPYNSVLSTHSLLEHIDVAVLLDNEAIYDICRRSLDIERPTY
Tua3_Eleusine_i SKLGFTIYPSPQVSTAVVEPYNSVLSTHSLLEHTDVAVLLDNEAIYDICRRSLDIERPTY
Tua1_Eleusine_i SKLGFTVYPSPQVSTSVVEPYNSVLSTHSLLEHTDVAVLLDNEAIYDICRRSLDIERPTY
Tua2_Eleusine_i SKLGFTIYPSPQISTAVVEPYNSVLSTHSLIEHTDVVVLLDNEAIYDICKRSLDIERPTY
TID1_Oryza_sati SKLGFTVYPSPQVSTSVVEPYNSVLSTHSLLEHTDVAVLLDNEAIYDICRRSLDIERPTY
Triticum_aestiv SKLGFTIYPSPQVSTAVVEPYNSVLSTHSLLEHTDVAVLLDNEAIYDICRRSLDIERPTY
Triticum_aestiv SKLGFTVYPSPQVSTSVVEPYNSVLSTHSLLEHTDVSILLDNEAIYDICRRSLDIERPTY
Prunus_mume_XP_ SKLGFTIYPSPQVSTAVVEPYNSVLSTHSLLEHTDVAVLLDNEAIYDICRRSLDIERPTY
Malus_domestica SKLGFTIYPSPQVSTAVVEPYNSVLSTHALLEHTDVSVLLDNEAIYDICRRSLDIERPTY
Populus_trichoc SKLGFTIYPSPQVSTAVVEPYNSVLSTHALLEHTDVTVLLDNEAIYDICRRSLDIERPTY
Ricinus_communi SKLGFTIYPSPQVSTAVVEPYNSVLSTHSLLEHTDVAVLLDNEAIYDICRRSLDIERPTY
Capsella_rubell SKLGFTIYPSPQVSTAVVEPYNSVLSTHSLLEHTDVAVLLDNEAIYDICRKSLDIERPTY
Glycine_max_NP_ SKLGFTIYPSPQVSTAVVEPYNSVLSTHSLLEHTDVAVLLDNEAIYDICRRSLDIERPTY
Ricinus_communi SKLGFTIFPSPQVSTAVVEPYNSVLSTHSLLEHTDVAVLLDNEAIYDICRKSLDIERPTY
Phoenix_dactyli SKLGFTIYPSPQVSTAVVEPYNSVLSTHSLLEHTDVAVLLDNEAIYDICRRSLDIERPTY
Phoenix_dactyli SKLGFTVYPSPQVSTSVVEPYNSVLSTHSLLEHTDVAVLLDNEAIYDICRRSLDIERPTY
Glycinemax_tua4 SKLGFTVYPSPQVSTSVVEPYNSVLSTHSLLEHTDVAVLLDNEAIYDICRRSLDIERPTY
Capsellarubella SKLGFTVYPSPQVSTSVVEPYNSVLSTHSLLEHTDVSILLDNEAIYDICRRSLSIERPTY
Ricinuscommunis SKLGFTVYPSPQVSTSVVEPYNSVLSTHSLLEHTDVAVLLDNEAIYDICRRSLDIERPTY
Malus_domestica SKLGFTVYPSPQVSTSVVEPYNSVLSTHSLLEHTDVAVLLDNEAIYDICRRSLDIERPTY
                ******..****.**.************.*.** ** .***********..** **** *
```

tid1 mutation in rice

kegne mutation in tef

Supplementary Fig. 9. Alignment of *α-Tubulin* genes in plants. The sequences of *α-Tubulin* genes were aligned to assess conservation across plant species. (Only a portion is shown.) At amino acid position 198, a nucleotide mutation of C to T causes an amino acid change of threonine (T) to isoleucine (I) in an otherwise conserved position. The alignment positions with the mutation of *kegne* in tef and *tid1* (*Twisted dwarf 1*) in rice are marked. The *kegne* mutation is highlighted in yellow. Positions marked with a star are identical in all sequences.

Supplementary Table 1. Yield and yield components of 312 WT and *kegne* plants grown in a tray of 0.128 m^2 under equal day conditions. 100-seed weight was calculated as average from 10 x 100 seeds. One standard deviation is given in parentheses. Yield per hectare was extrapolated from the harvest obtained from the tray.

Genotype	Total biomass (g/tray)	Seed yield (g/tray)	Straw yield (g/tray)	Harvest index (%)	100-seed weight (mg)	Estimated seed yield (t/ha)
WT	112.93	24.5	88.43	27.70	33 (SD 2.3)	1.91
Kegne	104.29	31.0	73.29	42.30	31 (SD 0.9)	2.42

References

Altschul SF, Gish W, Miller W, Myers EW, Lipman DJ. 1990. Basic Local Alignment Search Tool. *Journal of molecular biology* **215**, 403-410.

Bennetzen JL, Schmutz J, Wang H, Percifield R, Hawkins J, Pontaroli AC, Estep M, Feng L, Vaughn JN, Grimwood J, Jenkins J, Barry K, Lindquist E, Hellsten U, Deshpande S, Wang X, Wu X, Mitros T, Triplett J, Yang X, Ye CY, Mauro-Herrera M, Wang L, Li P, Sharma M, Sharma R, Ronald PC, Panaud O, Kellogg EA, Brutnell TP, Doust AN, Tuskan GA, Rokhsar D, Devos KM. 2012. Reference genome sequence of the model plant Setaria. *Nature biotechnology* **30**, 555-561.

Cannarozzi G, Plaza-Wuthrich S, Esfeld K, Larti S, Wilson YS, Girma D, de Castro E, Chanyalew S, Blosch R, Farinelli L, Lyons E, Schneider M, Falquet L, Kuhlemeier C, Assefa K, Tadele Z. 2014. Genome and transcriptome sequencing identifies breeding targets in the orphan crop tef (Eragrostis tef). *BMC genomics* **15**.

Chen IC, Huang IC, Liu MJ, Wang ZG, Chung SS, Hsieh HL. 2007. Glutathione S-transferase interacting with far-red insensitive 219 is involved in phytochrome A-mediated signaling in Arabidopsis. *Plant Physiology* **143**, 1189-1202.

Du H, Liu LH, You L, Yang M, He YB, Li XH, Xiong LZ. 2011. Characterization of an inositol 1,3,4-trisphosphate 5/6-kinase gene that is essential for drought and salt stress responses in rice. *Plant Molecular Biology* **77**, 547-563.

Goodstein DM, Shu S, Howson R, Neupane R, Hayes RD, Fazo J, Mitros T, Dirks W, Hellsten U, Putnam N, Rokhsar DS. 2012. Phytozome: a comparative platform for green plant genomics. *Nucleic acids research* **40**, D1178-D1186.

Guindon S, Lethiec F, Duroux P, Gascuel O. 2005. PHYML Online - a web server for fast maximum likelihood-based phylogenetic inference. *Nucleic acids research* **33**, W557-W559.

Hou CC, Tian W, Kleist T, He K, Garcia V, Bai FL, Hao YL, Luan S, Li LG. 2014. DUF221 proteins are a family of osmosensitive calcium-permeable cation channels conserved across eukaryotes. *Cell Research* **24**, 632-635.

Katoh K, Kuma K, Toh H, Miyata T. 2005. MAFFT version 5: improvement in accuracy of multiple sequence alignment. *Nucleic acids research* **33**, 511-518.

Kawahara Y, de la Bastide M, Hamilton JP, Kanamori H, McCombie WR, Ouyang S, Schwartz DC, Tanaka T, Wu JZ, Zhou SG, Childs KL, Davidson RM, Lin HN, Quesada-Ocampo L, Vaillancourt B, Sakai H, Lee SS, Kim J, Numa H, Itoh T, Buell CR, Matsumoto T. 2013. Improvement of the Oryza sativa Nipponbare reference genome using next generation sequence and optical map data. *Rice* **6**.

Lamesch P, Berardini TZ, Li D, Swarbreck D, Wilks C, Sasidharan R, Muller R, Dreher K, Alexander DL, Garcia-Hernandez M, Karthikeyan AS, Lee CH, Nelson WD, Ploetz L, Singh S, Wensel A, Huala E. 2012. The Arabidopsis Information Resource (TAIR): improved gene annotation and new tools. *Nucleic acids research* **40**, D1202-1210.

Larkin MA, Blackshields G, Brown NP, Chenna R, McGettigan PA, McWilliam H, Valentin F, Wallace IM, Wilm A, Lopez R, Thompson JD, Gibson TJ, Higgins DG. 2007. Clustal W and Clustal X version 2.0. *Bioinformatics* **23**, 2947-2948.

Paterson AH, Bowers JE, Bruggmann R, Dubchak I, Grimwood J, Gundlach H, Haberer G, Hellsten U, Mitros T, Poliakov A, Schmutz J, Spannagl M, Tang H, Wang X, Wicker T, Bharti AK, Chapman J, Feltus FA, Gowik U, Grigoriev IV, Lyons E, Maher CA, Martis M, Narechania A, Otillar RP, Penning BW, Salamov AA, Wang Y, Zhang L, Carpita NC, Freeling M, Gingle AR, Hash CT, Keller B, Klein P, Kresovich S, McCann MC, Ming R, Peterson DG, Mehboob-ur-Rahman, Ware D, Westhoff P, Mayer KFX, Messing J, Rokhsar DS. 2009. The Sorghum bicolor genome and the diversification of grasses. *Nature* **457**, 551-556.

Rambaut A. FigTree. Vol. 2014.

Sakai H, Lee SS, Tanaka T, Numa H, Kim J, Kawahara Y, Wakimoto H, Yang C, Iwamoto M, Abe T, Yamada Y, Muto A, Inokuchi H, Ikemura T, Matsumoto T, Sasaki

T, Itoh T. 2013. Rice Annotation Project Database (RAP-DB): An Integrative and Interactive Database for Rice Genomics. *Plant and Cell Physiology* **54**, E6-+.
Schnable PS, Ware D, Fulton RS, Stein JC, Wei FS, Pasternak S, Liang CZ, Zhang JW, Fulton L, Graves TA, Minx P, Reily AD, Courtney L, Kruchowski SS, Tomlinson C, Strong C, Delehaunty K, Fronick C, Courtney B, Rock SM, Belter E, Du FY, Kim K, Abbott RM, Cotton M, Levy A, Marchetto P, Ochoa K, Jackson SM, Gillam B, Chen WZ, Yan L, Higginbotham J, Cardenas M, Waligorski J, Applebaum E, Phelps L, Falcone J, Kanchi K, Thane T, Scimone A, Thane N, Henke J, Wang T, Ruppert J, Shah N, Rotter K, Hodges J, Ingenthron E, Cordes M, Kohlberg S, Sgro J, Delgado B, Mead K, Chinwalla A, Leonard S, Crouse K, Collura K, Kudrna D, Currie J, He RF, Angelova A, Rajasekar S, Mueller T, Lomeli R, Scara G, Ko A, Delaney K, Wissotski M, Lopez G, Campos D, Braidotti M, Ashley E, Golser W, Kim H, Lee S, Lin JK, Dujmic Z, Kim W, Talag J, Zuccolo A, Fan C, Sebastian A, Kramer M, Spiegel L, Nascimento L, Zutavern T, Miller B, Ambroise C, Muller S, Spooner W, Narechania A, Ren LY, Wei S, Kumari S, Faga B, Levy MJ, McMahan L, Van Buren P, Vaughn MW, Ying K, Yeh CT, Emrich SJ, Jia Y, Kalyanaraman A, Hsia AP, Barbazuk WB, Baucom RS, Brutnell TP, Carpita NC, Chaparro C, Chia JM, Deragon JM, Estill JC, Fu Y, Jeddeloh JA, Han YJ, Lee H, Li PH, Lisch DR, Liu SZ, Liu ZJ, Nagel DH, McCann MC, SanMiguel P, Myers AM, Nettleton D, Nguyen J, Penning BW, Ponnala L, Schneider KL, Schwartz DC, Sharma A, Soderlund C, Springer NM, Sun Q, Wang H, Waterman M, Westerman R, Wolfgruber TK, Yang LX, Yu Y, Zhang LF, Zhou SG, Zhu Q, Bennetzen JL, Dawe RK, Jiang JM, Jiang N, Presting GG, Wessler SR, Aluru S, Martienssen RA, Clifton SW, McCombie WR, Wing RA, Wilson RK. 2009. The B73 Maize Genome: Complexity, Diversity, and Dynamics. *Science* **326**, 1112-1115.
TAIR. The Arabidopsis Information Resource. Vol. 2014.
Thompson JD, Higgins DG, Gibson TJ. 1994. CLUSTAL W: improving the sensitivity of progressive multiple sequence alignment through sequence weighting, position-specific gap penalties and weight matrix choice. *Nucleic acids research* **22**, 4673-4680.
Toledo-Ortiz G, Huq E, Quail PH. 2003. The Arabidopsis basic/helix-loop-helix transcription factor family. *Plant Cell* **15**, 1749-1770.
van Nocker S, Ludwig P. 2003. The WD-repeat protein superfamily in Arabidopsis: conservation and divergence in structure and function. *BMC genomics* **4**.
Vogel JP, Garvin DF, Mockler TC, Schmutz J, Rokhsar D, Bevan MW, Barry K, Lucas S, Harmon-Smith M, Lail K, Tice H, Grimwood J, McKenzie N, Huo NX, Gu YQ, Lazo GR, Anderson OD, You FM, Luo MC, Dvorak J, Wright J, Febrer M, Idziak D, Hasterok R, Lindquist E, Wang M, Fox SE, Priest HD, Filichkin SA, Givan SA, Bryant DW, Chang JH, Wu HY, Wu W, Hsia AP, Schnable PS, Kalyanaraman A, Barbazuk B, Michael TP, Hazen SP, Bragg JN, Laudencia-Chingcuanco D, Weng YQ, Haberer G, Spannagl M, Mayer K, Rattei T, Mitros T, Lee SJ, Rose JKC, Mueller LA, York TL, Wicker T, Buchmann JP, Tanskanen J, Schulman AH, Gundlach H, de Oliveira AC, Maia LD, Belknap W, Jiang N, Lai JS, Zhu LC, Ma JX, Sun C, Pritham E, Salse J, Murat F, Abrouk M, Bruggmann R, Messing J, Fahlgren N, Sullivan CM, Carrington JC, Chapman EJ, May GD, Zhai JX, Ganssmann M, Gurazada SGR, German M, Meyers BC, Green PJ, Tyler L, Wu JJ, Thomson J, Chen S, Scheller HV, Harholt J, Ulvskov P, Kimbrel JA, Bartley LE, Cao PJ, Jung KH, Sharma MK, Vega-Sanchez M, Ronald P, Dardick CD, De Bodt S, Verelst W, Inze D, Heese M, Schnittger A, Yang XH, Kalluri UC, Tuskan GA, Hua ZH, Vierstra RD, Cui Y, Ouyang SH, Sun QX, Liu ZY, Yilmaz A, Grotewold E, Sibout R, Hematy K, Mouille G, Hofte H, Pelloux J, O'Connor D, Schnable J, Rowe S, Harmon F, Cass CL, Sedbrook JC, Byrne ME, Walsh S, Higgins J, Li PH, Brutnell T, Unver T, Budak H, Belcram H, Charles M, Chalhoub B, Baxter I, Initiative IB. 2010. Genome sequencing and analysis of the model grass Brachypodium distachyon. *Nature* **463**, 763-768.

Wang H, Hill K, Perry SE. 2004. An Arabidopsis RNA lariat debranching enzyme is essential for embryogenesis. *Journal of Biological Chemistry* **279**, 1468-1473.
